# Deep learning versus hybrid regularized extreme learning machine for multi-month drought forecasting: A comparative study and trend analysis in tropical region

**DOI:** 10.1016/j.heliyon.2023.e22942

**Published:** 2023-11-28

**Authors:** Mohammed Majeed Hameed, Siti Fatin Mohd Razali, Wan Hanna Melini Wan Mohtar, Majed Omar Ahmad Alsaydalani, Zaher Mundher Yaseen

**Affiliations:** a Green Engineering and Net Zero Solution (GREENZ), Department of Civil Engineering, Faculty of Engineering and Built Environment, Universiti Kebangsaan Malaysia (UKM), 43600 UKM, Bangi, Selangor, Malaysia; b Department of Civil Engineering, Al-Maarif University College, 31001, Ramadi, Iraq; c Smart and Sustainable Township Research Centre (SUTRA), Universiti Kebangsaan Malaysia (UKM), 43600, UKM, Bangi, Selangor, Malaysia; d Department of Civil Engineering, Umm Al-Qura University, Makkah, Saudi Arabia; e Civil and Environmental Engineering Department, King Fahd University of Petroleum & Minerals, 31261, Dhahran, Saudi Arabia; f Interdisciplinary Research Centre for Membranes and Water Security, King Fahd University of Petroleum & Minerals, 31261, Dhahran, Saudi Arabia

**Keywords:** Drought forecasting, Innovative trend analysis, Deep learning, Climate smart agriculture, Drought turning points, Multivariate standardized streamflow index

## Abstract

Drought is a hazardous natural disaster that can negatively affect the environment, water resources, agriculture, and the economy. Precise drought forecasting and trend assessment are essential for water management to reduce the detrimental effects of drought. However, some existing drought modeling techniques have limitations that hinder precise forecasting, necessitating the exploration of suitable approaches. This study examines two forecasting models, Long Short-Term Memory (LSTM) and a hybrid model integrating regularized extreme learning machine and Snake algorithm, to forecast hydrological droughts for one to six months in advance. Using the Multivariate Standardized Streamflow Index (MSSI) computed from 58 years of streamflow data for two drier Malaysian stations, the models forecast droughts and were compared to classical models such as gradient boosting regression and K-nearest model for validation purposes. The RELM-SO model outperformed other models for forecasting one month ahead at station S1, with lower root mean square error (*RMSE* = 0.1453), mean absolute error (*MAE* = 0.1164), and a higher Nash-Sutcliffe efficiency index (*NSE* = 0.9012) and Willmott index (*WI* = 0.9966). Similarly, at station S2, the hybrid model had lower (*RMSE* = 0.1211 and *MAE* = 0.0909), and higher (*NSE* = 0.8941 and *WI* = 0.9960), indicating improved accuracy compared to comparable models. Due to significant autocorrelation in the drought data, traditional statistical metrics may be inadequate for selecting the optimal model. Therefore, this study introduced a novel parameter to evaluate the model's effectiveness in accurately capturing the turning points in the data. Accordingly, the hybrid model significantly improved forecast accuracy from 19.32 % to 21.52 % when compared with LSTM. Besides, the reliability analysis showed that the hybrid model was the most accurate for providing long-term forecasts. Additionally, innovative trend analysis, an effective method, was used to analyze hydrological drought trends. The study revealed that October, November, and December experienced higher occurrences of drought than other months. This research advances accurate drought forecasting and trend assessment, providing valuable insights for water management and decision-making in drought-prone regions.

## Highlights

• Trend analysis and drought forecasting up to 6 months in advance are conducted
• Deep learning and hybrid models compared for drought forecasting in Malaysia
• Introducing novel parameter based on turning points to enhance the evaluation process
• Hybrid model enhances forecast accuracy by 21.52 % compared to LSTM.
• Trend analysis shows increased droughts in October, November, and December.

## Graphical abstract

**Figure:**
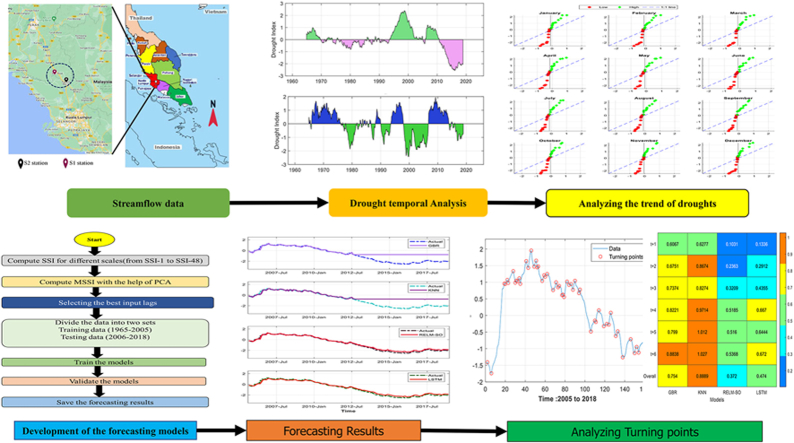

## 1 Introduction

Drought, a devastating natural calamity, is defined by an extended period of below-normal precipitation. It ranks among the most severe worldwide catastrophes due to its far-reaching consequences and substantial economic losses. Droughts are responsible for 22 % of economic damages resulting from disasters on a global scale, and they account for 33 % of the harm concerning the people impacted [1,2]. Typically, drought is divided into four primary forms: meteorological, hydrological, agricultural, and socio-economic drought. Drought is considered a gradual process that begins with a shortfall in precipitation, known as meteorological drought. An extended meteorological drought may cause a hydrological drought characterized by reduced dam reservoir capacity, decreased river flow, and lower lake water levels. Furthermore, extended droughts can trigger crop-threatening water shortages, hindering growth and development due to inadequate water supply.

Various drought indices developed since the 1960s based on meteorological and hydrological variables to identify and quantify drought. The “standardized precipitation index” (SPI) [3] is widely used for drought quantification, while the surface water supply index (SWSI) [4], standardized runoff index (SRI) [5], and standardized streamflow index (SSI) [6,7] are more famous indices used for describing the hydrological drought. The SSI is more popular than the SWSI index because it's easier to use and requires only streamflow data, whereas determining the variable weights of SWSI is subjective [8] and demands lots of input data (e.g., temperature, streamflow, rainfall, reservoir storage levels, and snow). Moreover, the ability of SSI to provide precise assessments of hydrological droughts is similar to the evaluations carried out by SWSI [9].

Hydrological drought forecasting can aid decision-making, early warning and mitigation strategies, but it is challenging due to complexity and creeping nature (slow progression). Drought forecasting involves selecting appropriate models, including physical, conceptual, and artificial intelligence (AI)-based models’ models. While physical and conceptual models are data-intensive and consider the process of a studied catchment [10], AI models are cost-effective models and require minimal data. Various models for drought forecasting have been used in literature, including artificial neural networks [11,12], support vector regression [13,14], and assembling models (e.g., random forest, gradient boost regression) [15,16]. Furthermore, some scholars have turned to the extreme learning machine (ELM) as an upgraded version of the traditional neural network. While conventional neural networks have some drawbacks, such as slow performance and poor generalization, the ELM has shown promising results in achieving good forecasting accuracy, particularly in forecasting droughts [[17], [18], [19]].

Developing models to obtain highly accurate predictions continues to be a primary focus of researchers. It is important to note that researchers are pursuing two main trends in using advanced AI models for drought forecasting. The first trend involves utilizing Metaheuristic algorithms (e.g., whale optimization algorithm, butterfly optimization, ant colony algorithm, imperialistic competitive algorithm, firefly optimization algorithm, genetic algorithm, and others) to enhance the performance of classical models and create hybrid models that eventually offer superior forecasting accuracy [10,[20], [21], [22], [23], [24], [25], [26], [27], [28], [29]]. These models have been shown to be highly effective in forecasting droughts compared to classical models. Another direction that researchers have taken is the broad application of advanced deep learning algorithm (e.g., long short-term memory (LSTM)), which can process large amounts of data and identify complex patterns [[30], [31], [32], [33], [34], [35], [36], [37]]. This approach has proven to be highly effective in generating accurate drought predictions. Deep learning and hybrid models are promising for drought forecasting, but more evaluation and comparison are needed to understand their capabilities and limitations.

Understanding drought trends is crucial for developing effective strategies to mitigate the negative impacts of drought. This knowledge is useful in water resource management, crop production, disaster risk reduction, and long-term adaptation planning. There are various methods to analyze drought patterns and trends, including non-parametric and parametric methods like the “Mann-Kendall test, Sen's slope, Spearman's rho test, and linear regression” [[38], [39], [40], [41], [42]]. However, these methods have restrictive assumptions [43], and the presence of positive serial correlation in time series data can increase the probability of identifying a trend even when there is none [44]. To address this issue, the pre-whitening technique was proposed, but it can remove a significant portion of data trend [45,46]. To solve this problem, the innovative trend analysis (ITA) method was proposed by Ref. [47], which does not impose any restrictive assumptions and has shown greater effectiveness than traditional methods in identifying drought trends (e.g., such as serial correlation or seasonal cycles) [48]. ITA has been successfully used in diverse climatic region in identifying drought trends [[49], [50], [51], [52], [53]].

The primary objective of this research is to enhance the understanding of drought trend dynamics and provide reliable forecasts for droughts several months in advance. This knowledge is crucial for improved water management planning and for establishing efficacious advanced early warning systems and drought policy preparations. Previous research efforts have primarily focused on either forecasting drought using predictive models or analyzing drought patterns and trends. However, they did not integrate both methods concurrently, creating a gap in the comprehensive understanding of drought dynamics. Therefore, this research aims to bridge that divide by combining the analysis of drought patterns and trends with predictive models. The study includes a comprehensive analysis of popular and robust forecasting models in the field of drought, such as DL and hybrid models. This aspect of the research enhances the depth of the investigation, mainly because previous studies have not explored such a comparison between the two forecasting models. The Multivariate Standardized Streamflow Index (MSSI) is adopted, considering all drought time scales in contrast to indices like SDI and SSI [54]. Thus, the performances of a hybrid model that combines Regularized Extreme Learning Machine (RELM) with a novel snake optimizer (RELM-SO) and a Deep Learning Model (LSTM) are investigated. The models forecast drought for one to six months in advance at two specifically dry locations in Selangor State, Malaysia [55]. Also, the models are validated against benchmark standalone models, such as k-nearest neighbor and gradient boosting regression, as well as other robust models developed in the literature. Typically, the traditional statistical metrics may be inadequate for evaluating the effectiveness of drought forecasting models due to the high autocorrelation in time series data [[56], [57], [58]], and the issue remains a challenge not addressed in previous studies [11,59]. Consequently, this study validates models based on their ability to accurately capture critical and turning points (i.e., critical points indicate significant changes/events, while turning points signal trend direction shifts in drought time series data), aiming to identify the most effective model for reliable drought forecasting. Finally, this research also comprehensively analyses drought trends for all months of the year. The ITA method is utilized to identify the most vulnerable months for severe drought, as it is an effective tool that does not require numerous variables and assumptions.

## 2 Statistical methods and case study

### 2.1 Multivariate standardized streamflow index (MSSI)

The calculation of MSSI involves multiple SSI time series for different aggregation time scales (K) at specific stations. While studying various time scales in classical indices such as SSI can provide information on different types of droughts, it may also cause confusion due to its complexity [54]. Therefore, to derive MSSI, it is suggested to reduce the number of SSI time scales and summarize the variability using principal components analysis (PCA). MSSI is derived PCA to a set of *K* time series of SSI, with *K* representing the drought time scale of SSI. *PC*_*1*_, the first principal component of the PCA analysis, holds essential information on the percentage of variation in *K* for the initial variable. As a result of the characteristics of *PC*_*1*_*,* its values cannot be compared between months, unlike SSI, which has a specific statistical characteristic (mean is 0 and a standard deviation is 1). Hence, to standardize the *PC*_*1*_ time series, it is essential to use the average and standard deviation of different months throughout the year [54,60]. Notably, Equation (1) provides the mathematical formula that used for standardizing the drought data.

(1) $$Z_{1ym}=\frac{{PC}_{1ym}\;-\bar{{PC}_{1m}}}{{SD}_{1m}}$$

In the above formula, the $Z_{lym}$ refers to the standardized value of ${PC}_{1}$ in year y and month m, The term ${PC}_{1ym}$ is the *PC*_*1*_ value in *y*th year an *m*th month, $\bar{{PC}_{1m}}$ , ${SD}_{1m}$ and are the mean and standard deviation of the *PC*_*1*_ of the mth month. Notably, $Z_{1ym}$ is considered as the MSSI. Since the $\bar{{PC}_{1m}}$ value is statistically insignificant and almost zero, it can be ignored in the equation's numerator. The MSSI time series as represented in $Z_{1ym}$ is organized in ascending order, and a graphic of its empirical probability distribution is shown to identify the categories of drought and wet severity (Appendix A).

### 2.2 Case study location

**Fig. 1:**
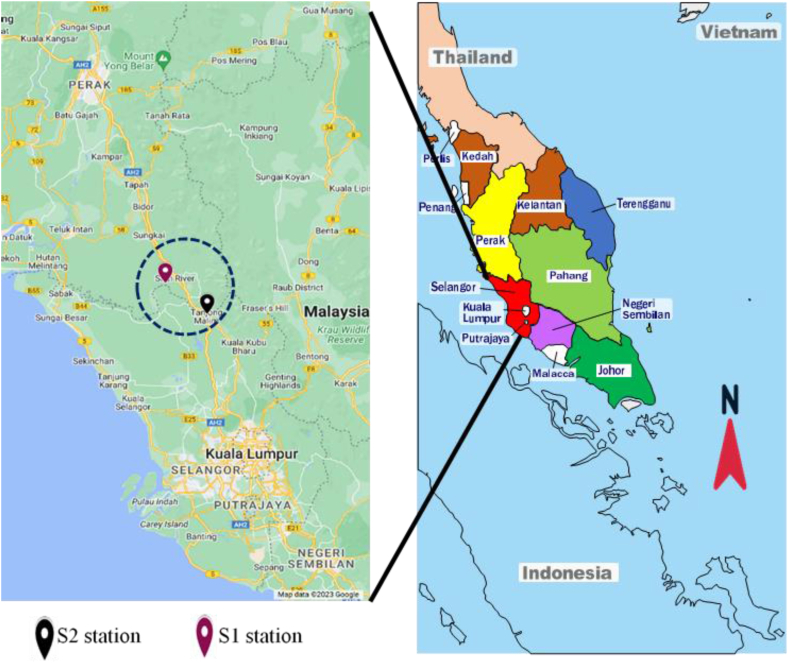
The illustrates the geographic locations of the streamflow stations investigated in this study.

The daily streamflow records utilized in this research were collected from two stations located in Selangor, Malaysia, spanning an extensive period from 1961 to 2018. The first station (S1) is located in Bernam River, which covers an area of 3335 km^2^ and is a crucial region for agriculture and water supply for Selangor and Perak, mainly for irrigation [61]. Besides, the river basin has relatively high temperatures, humidity, and an average annual precipitation varies between 2000 and 3500 mm. Recently, urbanization resulting from changes in the Malaysian government's economic policies has influenced water quality and quantity in the region. The second station (S2), is located in the Selangor River and has a catchment area of 2200 km^2^ [62]. It is a primary water source for several regional water treatment plants. The Selangor River basin has diverse natural landscapes, including forests, agricultural areas, and developed regions. The geographical locations of these studied streamflow stations are illustrated in Fig. 1.

The geographical coordinates and statistical characteristics of both study stations are summarized in Table 1. Station S1 has higher maximum, average, and minimum flow rates compared to station S2, with values of 204.7320 m^3^/s, 56.6064 m^3^/s, and 4.4790 m^3^/s, respectively, compared to 29.7832 m^3^/s, 8.9139 m^3^/s, and 1.2070 m^3^/s at station S2. However, station S1 also exhibits much higher variability in flow rate measurements than station S2, with a standard deviation of 30.1697 m^3^/s compared to 4.8189 m^3^/s at station S2.

**Table 1:** General information and statistical description of the studied streamflow stations.

Description	Streamflow stations	
	S1	S2
Longitude	101° 31″ 24′	101° 26″ 35′
Latitude	03° 40″ 67′	03° 24″ 10′
District	Hulu Selangor	Kuala Selangor
River Basin	Bernam	Selangor
Data duration	1-1-1961 to 31-1-2018	1-1-1961 to 31-1-2018
Maximum value	204.7320 m^3^/s	29.7832 m^3^/s
Average value	56.6064 m^3^/s	8.9139 m^3^/s
Minimum value	4.4790 m^3^/s	1.2070 m^3^/s
Standard deviation	30.1697 m^3^/s	4.8189 m^3^/s
Skewness	1.1593	1.1375
Station Code	S3615412	S3414421

**Fig. 2:**
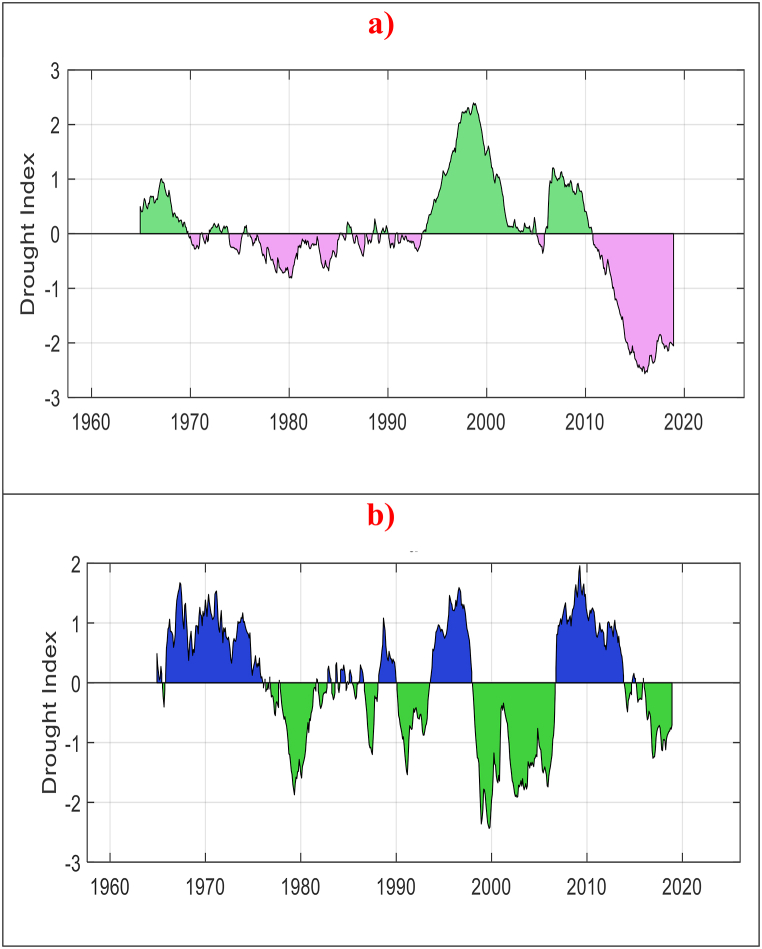
Temporal variation of MSSI for both hydrological stations. a) S1 station, and b) S2 station.

The MSSI was calculated using PCA, which integrated SSI data collected at various time intervals, ranging from SSI-1 to SSI-48. According to the hydrological drought depicted in Fig. 2 (a, and b), both selected stations experienced severe drought events between 1978 and 2018. Station S2 faced more than five such events, while S1 experienced only one during the same time period. It is worth highlighting that the S1 station experienced severe drought conditions for a longer period between 2010 and 2018. Overall, the presented result of hydrological drought provides compelling evidence that droughts are an issue in the region, with both stations being significantly impacted.

### 2.3 Trend analysis: Sen-innovative trend method

The innovative trend analysis method (ITAM) is a novel approach used to detect the trend of time-series data proposed by Şen [47]. The core concept of this approach involves dividing a time series into two equal parts spanning from the first date to the end date of the studied data. The two divided segments are sorted in ascending order and exhibited on the *X* and *Y*-axis, respectively. In the Cartesian coordinate system, both divided segments are sorted in ascending order and plotted on the *X* and *Y*-axis. The first segment (Xi:i=1,2,...,n/2) is plotted on the horizontal axis, while the second segment (Yj:j=n/2+1,n/2+2,...,n) is plotted on the vertical axis. Appendix B illustrates how a line bisects at 1:1 (45°) separates the diagram into two triangles of equal dimensions. According to Appendix B, an ascending trend is represented by the upper triangle, whereas a descending trend is depicted by the lower one. To estimate the trend, the slope of the Innovative trend analysis method called $S_{ITAM}$ statistic is calculated using Equation (2).

(2) $$S_{ITAM}=\frac{2\left(\bar{X_{i}}-\bar{Y_{i}}\right)}{n}$$

where, $\bar{X_{i}}$, and $\bar{Y_{i}}$ are the average value of the first and second half of the time-series data and n is the length of the data.

To determine if there is a statistically significant trend in a time series, the null hypothesis (*H*_*o*_) is rejected if the calculated slope value ($S_{ITAM}$) does not exceed a critical value ($S_{sci}$). On the other hand, if $S_{ITAM}$ > $S_{sci}$, the alternative hypothesis (*H*_*o*_) indicating the presence of a significant trend is applicable. The probability density function (*PDF*) of the null hypothesis is used to calculate the test significance, and the confidence limits (*CL*) of the trend slope can be derived by setting $S_{sci}$ as the confidence interval of a standard normal *PDF* with a mean of zero and a standard deviation of $\sigma_{s}$ at a significance level of α. Thus, the *CL* can be computed using Equation (3)

(3) $${CL}_{\left(1-a\right)}=0\;\mp S_{sci}\times \sigma_{s}$$

In the above equation, the $\sigma_{s}$ is slop standard deviation. Notably, the test details can be found in Refs. [47,63]. The ITAM was used in this study to assess the importance of monthly hydrological drought changes. A 5 % significance level was applied, considering the infrequency of significant changes in drought.

## 3 Applied artificial intelligence models

### 3.1 Long short-term memory (LSTM)

LSTM is one of the most popular applications of deep learning neural networks, which has been used widely in literature to model time series data [64]. It is considered an improved version of recurrent neural network (RNN). Capturing long-term time correlations can be problematic with a traditional RNN, which commonly encounters gradient disappearance (i.e., vanishing gradient) and explosion problems [65]. LSTM is a sort of time-cyclic neural network which implemented partially to address the long-term correlation problem of RNN [66,67]. Besides, it has good architecture enables the forecasting model to learn from training data across numerous time steps. Furthermore, the memory cell within the hidden layer neural nodes of the cyclic neural network can be utilized to anchor and retain past information. The model can effectively use the historical data by integrating three-active gate structures (i.e., input, forget, and output gates).

Based on Appendix C, the sequence input of drought time series data *x* (*x*_1_, *x*_2_, *x*_3_, …, *x*_t_) when inserted into the LSTM model, the hidden layer states (*h*_1_, *h*_2_, *h*_3_, …, *h*_*t*_) and the output of memory unit can be calculated as using Equations (4), (5), (6), (7), (8) [68]:

(4) $$i_{t}=sigmoid\left(W_{hi}h_{t-1}+W_{xi}x_{t}\right)$$

(5) $$f_{t}=sigmoid\left(W_{hf}h_{t-1}+W_{xf}x_{t}\right)$$

(6) $$c_{t}=f_{t}⊕c_{t-1}+i_{c}⊕\;\tanh\left(W_{xc}x_{t}+W_{hc}h_{t-1}\right)$$

(7) $$o_{t}=sigmoid\left(W_{xo}x_{t}+W_{ho}x_{t-1}+W_{co}c_{t}\right)$$

(8) $$h_{t}=o_{t}⊕\tanh\left(c_{t}\right)$$

In the above equations, the symbol ⊕ is Hadamard product; $c_{t}$ is the cell state's vector; $o_{t}$, $i_{t}$, and $f_{t}$ are representing the output of different gates; $h_{t-1}$ is the output data of the hidden layer unit regarding the previous iteration, while the new state of the memory cell is $h_{t}$; $W_{c}$, $W_{f}$, and $W_{h}$ are the corresponding gate's weights, and the tanh, and sigmoid are activation functions.

### 3.2 K-nearest neighbor (KNN)

KNN, a supervised machine learning approach, can be employed for conducting several tasks such as classification, clustering, and regression. Its algorithm is not complex and requires no assumptions on the primary distribution of the data. The KNN is a nonparametric model, so its concept depends mainly on similarity (neighborhood) measures. To forecast the query point of MSSI, KNN first starts to gather data observations close to that point. Notably, the neighbors of a query point can be calculated by several distance functions (e.g., Euclidean approach). Once nearby data points have been identified, the algorithm sorts them based on their distance from the new data point (query point). The next step involves selecting a specific number of data points with the shortest distances and assigning their responses. The data points are chosen according to the value of *k* (number of neighbors) that the user defined. Finally, assemble the responses of the closed k-points to forecast the response of the query point. For more details on the algorithm, please see the following reference [69].

### 3.3 Gradient boosting regression (GBR)

The GBR technique can be defined as a machine learning ensemble method which boosts the predictive accuracy of a conventional decision tree model. It is achieved by integrating boosting, a sequential statistical process that combines multiple weak predictive models to produce a single, highly precise model [70]. The method employs an iterative approach, refining the weak learner's tree model estimates by incorporating the pseudo residuals of the current learner, disregarding the negative gradient of the loss function [71]. The iteration process continues until the GBR model's loss function is minimized at the lowest level and hence, the prediction accuracy improves. The iterative learning procedure of GBR with *K* decision tree is explained as follows:

For a given training data $D=\{\left(x_{1},y_{1}\right),\left(x_{2},y_{2}\right),...,\left(x_{n-1},y_{n-1}\right),(\;x_{n},y_{n})\}$, the loss function can be derived using Equation (9)

(9) $$L\left(y,f\left(x\right)\right)={\left(y-f\left(x\right)\right)}^{2}$$

in this context, *L* denotes the loss function, which relies on the actual value *y*, while *x* represents the input values.

Step 1 Initialize the new tree model (weak learner) with an initial constant value, as presented in Equation (10)

(10) $$f_{o}\left(x\right)=\arg\;\min_{c}\sum_{i=1}^{N}L\left(y_{i},c\right)\;$$

in Eq. (10), *N* represents the total number of data points, and the parameter *c* is a constant value.

Step 2 set the iteration number *m* = 1,2,3, …, K

i. For *i* = 1,2, 3, …, N. The pseudo residuals of the ith training data are determined using Equation (11).

(11) $$r_{m,i\;}={-\left[\frac{\partial L\left(y,f\left(x_{i}\right)\right)}{\partial f\left(x_{i}\right)}\right]}_{f\left(x\right)=f_{m-1}\left(x\right)}$$

$r_{m,i\;}$ is refers to the pseudo-residual associated with the *i*th training data point at the *m*th iteration of the model.

ii. Build a regression tree based on $r_{l,i\;}$ and determine the size of the leaf node, $R_{m,l\;}$, for the $m^{th}$ tree. To obtain an approximate value of the fitting residual, conduct a prediction for the area of the leaf nodes in the decision tree.
iii. Perform linear search for each value of *l* ranging from 1 to L within the leaf node range and minimize the loss function using gradient descent. The best fitting residual fitting values for each blade, resulting from the analysis, are presented in Equation (12).

(12) $$c_{m}=\arg\;\min_{c}\sum_{i=1}^{N}L\left(y_{i},f_{m-1}\left(x_{i}\right)+c\right)$$

Where, $c_{m}$ is the best residual fitting value for *m*th tree [70]

iv. Update the regression tress Using Equation (13).

(13) $$f_{m}\left(x\right)=f_{m-1}\left(x\right)+\sum_{l=1}^{L}c_{ml}I\left(x\in R_{ml}\right)$$

Step 3 Obtain the final model using Equation (14).

(14) $$f\left(x\right)=f_{M}\left(x\right)=\sum_{m=1}^{M}\sum_{l=1}^{L}c_{ml}I\left(x\in R_{ml}\right)$$

### 3.4 Regularized extreme learning machine (RELM)

ELM is one of the most famous types of Feed-Forward Networks with a Single Hidden Layer introduced by Ref. [72]. According to various sources, ELM has a straightforward structure, quick training time, strong generalization capabilities, and effective performance [[73], [74], [75]]. The principles of ELM training and prediction are detailed in Refs. [76,77]. In training ELM, the primary goal is to determine the weight vector (β) that connects the hidden layer and output layer, which is accomplished through the calculation of β. The computed weight vector (β) is given by Equation (15) below.

(15) $$\hat{\beta }=H^{†}T$$

In the above formula, the Moore-Penrose generalized inverse of the hidden layer output matrix *H* is denoted by $H^{†}$. Notably, the term $H^{†}$ is computed using the expression ${\left(H^{T}\times H\right)}^{-1}$ × $H^{T}$, where *T* represents the actual data vector.

Once the structural parameters have been determined, the trained model can forecast the output values. ELM's main flaw, however, is that it is unstable and prone to overfitting and interference from data noise and outlier values [78,79]. To address this issue, some researchers [80] presented a regularization parameter while determining the weight β, significantly improving the ELM model's generalization capability and practicality. As per statistical learning theory, the risk associated with prediction during the learning process is a combination of structural and empirical risks. A model with solid generalization capabilities should efficiently balance these two risks excellently. Consequently, the overall risk is represented as a weighted sum of the mentioned risks, where the ratio of empirical risks can be adjusted by introducing a weighting parameter γ [80]. Empirical risk can be computed using the squared error ${\left\|\varepsilon \right\|}^{2}$, while the variables $\|\beta {\|}^{2}$ that maximize the distance to the interface are used to represent the structural risk [81]. The mathematical formulation of the regularized extreme learning machine (RELM) model is presented in Equation (16):

(16) $$\left\{ \begin{array}{c} \min\;\left({\frac{1}{2}\left\|\beta \right\|}^{2}+\frac{1}{2}\gamma \|\varepsilon {\|}^{2}\right)\\ s.t.\sum_{i=1}^{N}\beta_{i}g\left(w_{i}\;x_{j}+b_{i}\right)-T_{j}\\ j=1,2,3,...,\tilde{N} \end{array}\right.$$

In the above formula, the *N* is the total number of neurons in the models' hidden layer, while the $\tilde{N}$ is the number of observations. Moreover, the terms j, w,b and x , T are the j th observation, wights, bias of hidden layer, the input and output data, respectively. The optimization problem presented in the equation above can be solved by creating the Lagrangian equation as illustrated in Equation (17).

(17) $$L\left(\beta ,\varepsilon ,a\right)=\left\{ \begin{array}{c} \frac{1}{2}\left\|\beta {\|}^{2}+\frac{1}{2}\gamma \right\|\varepsilon {\|}^{2}-\sum_{j=1}^{\tilde{N}\;}a_{j}\left(\sum_{i=1}^{N}\beta_{i}g\left(w_{i}\;x_{j}+b_{i}\right)-t_{j}\right)-\varepsilon_{j}\\ =\frac{1}{2}\left\|\beta {\|}^{2}+\frac{1}{2}\gamma \right\|\varepsilon {\|}^{2}\;-a\left(H\beta -T-\varepsilon \right) \end{array}\right.$$

The $a_{j}$ s the Lagrangian multiplier, which is presented in the above equation. The optimal condition for Karush-Kuhn-Tucker (KKT), which applies to L(β,ε,a), can be expressed in Equation (18).

(18) $$\left\{ \begin{array}{c} \frac{\partial L}{\partial \beta }=0\;\Rightarrow \beta^{T}=aH\\ \frac{\partial L}{\partial \varepsilon }=0\;\Rightarrow {\gamma \varepsilon }^{T}+a=0\\ \frac{\partial L}{\partial a}=0\;\Rightarrow H\beta -T-\varepsilon =0 \end{array}\right.$$

By combining the equations provided above, the final expression for β can be calculated using Equation (19).

(19) $$\hat{\beta }={\left(H^{T}H+\frac{I}{\gamma }\right)}^{-1}H^{T}T$$

When using the equation listed above to determine the weight vector $\hat{\beta }$ between the hidden layer and output layer, the resulting ELM is known as a RELM. By adjusting the value of γ, which represents the trade-off between empirical and structural risks, the model can achieve an optimal balance between these two risks [80]. Compared to the traditional ELM, the RELM model exhibits superior anti-interference ability, more robust neural network generalization, and higher prediction accuracy [82].

### 3.5 Snake optimizer (SO)

SO is a metaheuristic and sophisticated algorithm suggested by Ref. [83] and imitates the mating actions of snakes. The algorithm is triggered by conditions similar to those that activate the snake mating, which occurs when there is enough food, and the temperature is low. Like other metaheuristic methods, the SO algorithm generates random candidate solutions. Moreover, the swarms are divided equally into male and female groups. To determine the optimal candidate solution during each iteration, the SO algorithm analyzes each group to identify the best male and female individuals. The parameters for Temperature (T) and Food Quantity (FQ) can be explained in Equation (20), (21) [84].

(20) $$T=\exp\left(-\frac{g}{T}\right)$$

(21) $$FQ=C_{1}\;\exp\left(\frac{g-T}{T}\right)$$

where the term g refers to the current iteration, while T is the total iterations, and $C_{1}$ is a constant value ($C_{1}$ = 0.5). Thus, if FQ less than $C_{1}$, the snakes employ a food search approach in which they randomly select a position and subsequently update their current location. The mathematical model for the exploration step for both male and female snakes is illustrated below:

a) Male snakes: The exploration step for male is expressed using Equation (22).

(22) $$x_{i,j}\left(g+1\right)=x_{\left({rand}_{\in \left[1,N/2\right]},j\right)}\left(g\right)\mp C_{2}\times A_{i,male}\left(\left(ub-lb\right)\times {rand}_{\in u\left(0,1\right)}+lb\right)$$

where, $A_{i,male}=\exp\left(-\frac{f_{rand,male}}{f_{i,mail}}\right)$, and $C_{2}$ is a constant

In the above formula, N is the number of individuals, a male snake's location is $x_{i,j}$ while the $x_{\left({rand}_{\in \left[1,N/2\right]},j\right)}$ is the location of random male snake, and rand is a random number between zero and one. The fitness function of the previously nominated male snake for random search can be represented by $f_{rand,male}$, while $f_{i,mail}$ is the fitness function of *i*th male in the group. Finally, the upper and lower bounds are ub,andlb, respectively. The diversity factor, represented by the flag direction operator ±, is utilized to randomly explore all potential directions in the provided search space model.

b) Female snakes: The exploration step for Female is expressed using Equation (23).

(23) $$x_{i,j}=x_{\left({rand}_{\in \left[1,N/2\right]},j\right)}\left(g+1\right)\mp C_{2}\times A_{i,female}\left(\left(ub-lb\right)\times {rand}_{\in u\left(0,1\right)}+lb\right)$$

where, $A_{i,female}=\exp\left(-\frac{f_{rand,female}}{f_{i,female}}\right)$.

In the exploitation phase, the SO algorithm utilizes two conditions to determine the optimal solutions.

➢ If FQ > Threshold (i.e., T is more than 0.6), the snakes move to search for food only. Thus, the exploitation phase can be represented using Equation (24)

(24) $$x_{i,j}\left(g+1\right)=x_{food}\mp C_{3}\times T\times rand\times \left(x_{food}\;-x_{i,j}\left(g\right)\right)$$

The variable $x_{i,j}$ represents the location or status of individuals, regardless of gender (male or female)., $C_{3}$ is a constant, and $x_{food}$ is the position of the best individuals

➢ If FQ is less than the specified Threshold value (less than 0.6), The snakes will exhibit two distinct behaviors, which are either fighting or mating.

These behaviors can be illustrated using the following models for fighting and mating.

i. Fighting mode: the fighting capability of the male agent ($F_{\;male}$) is expressed in Equation (25) below:

(25) $$x_{i,j}\left(g+1\right)=x_{i,j}\left(g\right)+C_{3}\times F_{i,male}\times rand\times \left(x_{best,femail}-x_{i,male}\left(g\right)\right)$$

Where, $F_{i,male}=\exp\left(-\frac{f_{best,female}}{f_{i}}\right)$.

The term $x_{i,j}$ is the $i^{th}$ male position, $x_{best,femail}$ is the position of the best individual in the female group. Likewise, the male agent's fighting capability, $F_{i,male}$, can be expressed in Equation (26).

(26) $$x_{i,j}\left(g+1\right)=x_{i,j}\left(g\right)+C_{3}\times F_{i,female}\times rand\times \left(x_{best,male}-x_{i,female}\left(g+1\right)\right)$$

where, $F_{i,female}=\exp\left(-\frac{f_{best,male}}{f_{i}}\right)$.

ii. Mating mode: during the mating mode, both male and female agents possess the capability to modify their positions according to the following criteria (see Equation (27), (28)):

(27) $$x_{i,male}\left(g+1\right)=x_{i,m}\left(g\right)\mp C_{3}\times {MM}_{i,male}\times \left(FQ\times x_{i,female}-x_{i,male}\left(g\right)\right)$$

where, ${MM}_{i,male}$ = $\exp\left(-\frac{f_{i,female}}{f_{i},male\;}\right)$.

(28) $$x_{i,female}\left(g+1\right)=x_{i,f}\left(g\right)\mp C_{3}\times {MM}_{i,female}\times \left(FQ\times x_{i,male}-x_{i,female}\left(g+1\right)\right)$$

where, ${MM}_{i,female}$ = $\exp\left(-\frac{f_{i,male}}{f_{i},female\;}\right)$.

In the above equation, the $x_{i,f}$ , and $x_{i,m}$ are the positions of *i*th females and males, respectively. The ${MM}_{i,female}$ and ${MM}_{i,male}$ are the mating ability of females and males.

### 3.6 Developing the forecasting models

**Fig. 3:**
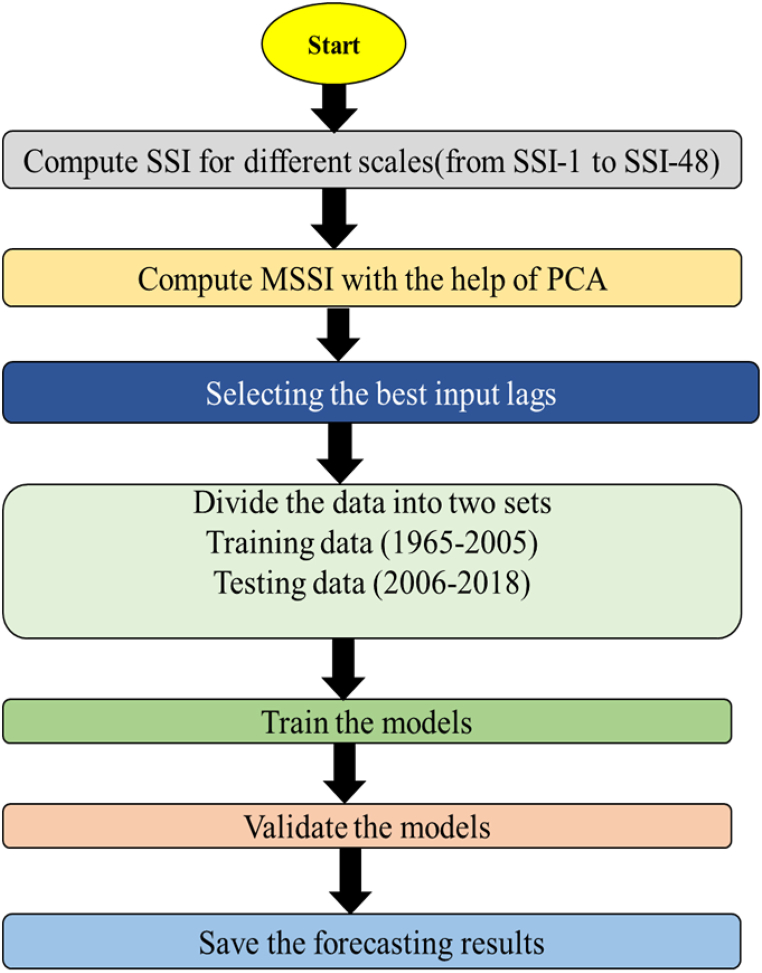
Block diagram the main processes that used for establishing the forecasting models.

In the current study, the applied models included KNN, GBR, and LSTM, along with a fourth hybrid model called (RELM-SO) that combined RELM and SO algorithms. The SO algorithm was used to optimize the RELM's parameters such as wight and bias values as well as regularization factor. Furthermore, this work uses root mean square error (RMSE) as the objective function, with the aim of minimizing it using the SO algorithm. The study's training methodology involved selecting optimal input lags via the partial autocorrelation function (Appendix D), splitting data into training and testing sets (75 % for training, and 25 % of testing), normalizing the data, initializing model parameters, and validating the models using several statistical metrics. Fig. 3 depicts the main process employed to develop the forecasting models, as illustrated in the accompanying flowchart. Notably, the candidate hyperparameters of each model are provided in Table 2.

**Table 2:** The candidate parameters of the applied models.

Models	Hyperparameters range
KNN	K = [2–200]
GBR	Learning rate = [0.0856–0.9670] Number of trees = [3–56]
LSTM	Number of Hidden Nodes = [80−260] Max-Epochs = [100−500] Gradient Threshold = [0.001–0.952] Initial Learn Rate = [0.0001–0.01] Learn Rate Drop Period = [100−300] Learn Rate Drop Factor = [0.3–1]
RELM-SO	Hidden nodes = [2–15] Maximum Iteration = [50−650] Search Agents = [8–120] Regularized parameter = [1/N, N]. Where N is the total number of training observations.

### 3.7 Statistical performance metrics

The study used the root mean square error (*RMSE*), Nash-Sutcliffe efficiency index (*NSE*), Mean absolute error (*MAE*), Willmott index (*WI*), and Correlation of determination coefficient (*R*^*2*^) as the five most commonly used statistical parameters to evaluate the effectiveness of the developed MSSI forecasting models. These parameters were expressed mathematically in the study using Equations (29), (30), (31), (32) [85,86].

i. Root mean square error (*RMSE*)

(29) $$RMSE=\sqrt{\frac{1}{n}\sum_{i=1}^{n}{\left({MSSI}_{{obs}_{i}}-{MSSI}_{{pred}_{i}}\right)}^{2}\;}$$

ii. Nash-Sutcliffe efficiency index (*NSE*)

(30) $$NSE=1-\frac{\sum_{i=1}^{n}\left|{MSSI}_{{obs}_{i}}-{MSSI}_{{pred}_{i}}\right|\;}{\sum_{i=1}^{n}\left|{MSSI}_{{obs}_{i}}-\bar{{MSSI}_{obs}}\right|}$$

iii. Correlation of determination (*R*^*2*^)

(31) $$R^{2}=1-\frac{\sum_{i=1}^{n}{\left({MSSI}_{{obs}_{i}}-{MSSI}_{{pred}_{i}}\right)}^{2}\;}{\sum_{i=1}^{n}{\left({MSSI}_{{pred}_{i}}-\bar{{MSSI}_{pred}}\right)}^{2}}$$

iv. Willmott index (*WI*)

(32) $$WI=1-\frac{\sum_{i=1}^{n}{\left({MSSI}_{{obs}_{i}}-{MSSI}_{{pred}_{i}}\right)}^{2}}{\sum_{i=1}^{n}{\left(\left|{MSSI}_{{pred}_{i}}-\;\bar{M{SSI}_{obs}}\right|+\left|{MSSI}_{{obs}_{i}}-\;\bar{{MSSI}_{obs}}\right|\right)}^{2}\;}$$

${MSSI}_{{obs}_{i}}$, and ${MSSI}_{{pred}_{i}}$ are observed and forecasted values, while n is the dataset's total number of drought values. The mean value for forecasted and calculated droughts are $\bar{{MSSI}_{pred}}$ , and $\bar{{MSSI}_{obs}}$. For the model to be considered optimal, it should produce error measures (*RMSE*, and *MAE*) that are as low as possible (approaching zero) and anticipate outcomes that closely match the measured values. Specifically, the values of *R*^*2*^, *WI*, and *NSE* should be closer to one, indicating a greater degree of consistency between the predicted and actual values.

### 3.8 Identification and analysis of turning points in drought time series data

In time series data, turning points are points where the trend of the data changes direction, while critical points are points where a significant change occurs, such as a sudden spike or drop in the values. Both types of points can be identified using various statistical techniques such as time series regression analysis. Turning points indicate changes in the underlying pattern of the data and can help identify shifts in the behavior of the phenomenon being studied. Critical points, on the other hand, can indicate the occurrence of an event or a change in the underlying process generating the data. These points may offer valuable insights into the dynamics of the system under study and can facilitate informed decision-making across a range of fields, including water resources planning and management.

The study assessed drought forecasting models using performance metrics. However, conventional indicators like *RMSE, R*^*2*^, and others may not be effective due to high auto-correlation in the drought time series data [[56], [57], [58]]. Thus, most forecasting models provide very high accuracy with a correlation coefficient of more than 0.9 [87]. Therefore, the study focused on identifying critical and turning points in time series data to evaluate the model's ability to simulate them accurately. The process involved detecting the turning points of original time series data (MSSI) and collecting corresponding values from multiple models, and then calculating the average absolute error of the turning points (*AAETP*) for each model separately. This process was conducted for both stations to assess the model's predictability in forecasting hydrological drought for six months in advance. In this study, the *AAETP* is expressed using Equation (33)

(33) $$AAETP=\text{average}\;\left(\left|E_{i}\right|\right)$$

The computation of $E_{i}$ (forecasting error computed for turning and critical points) for each model results in a vector is carried out by subtracting the forecasted values of the critical points (i.e., forecasted ones) from their corresponding actual values. The algorithm for computing turning points in a drought time series data can be summarized as follows:

i. Calculate the first derivative of the time series data (MSSI data) by taking the difference between consecutive data points.
ii. Find the sign of the first derivative using the sign function. The sign indicates whether the data is increasing or decreasing.
iii. Find the locations where the sign of the first derivative changes from positive to negative or vice versa. These locations are the turning points.
iv. Find the turning points by looking for the points where the absolute difference in signs is equal to 2 (i.e., second derivative).
v. Visualize the data and the turning points using a plot.
vi. Once turning points are identified, compute AAETP.

## 4 Result and discussions

### 4.1 Drought trend analysis results

The present study utilized monthly streamflow data spanning 58 years (1961–2018) to calculate the MSSI for two study stations. To determine MSSI, PCA combined different time scales of SSI, including SSI-1 up to SSI-48. The temporal distribution of the MSSI is presented in Fig. 2 (a, and b). Additionally, the trend analysis was conducted using the ITAM at a significant level of 5 %. One of the most notable features of ITAM is its ability to visually explore the trend of the time series data, whether increasing or decreasing. However, due to the fluctuating nature of drought data, using traditional methods to explore drought patterns can be challenging. To illustrate this point, let's take the S1 station as an example and analyze drought patterns for each month separately, as shown in Appendix E. At first glance, it appears that every month has a dry period followed by a wet period, making it difficult to discern the drought trend. Therefore, this study adopted a technique of separating low drought values from high values. The monthly MSSI data is divided into two groups based on optimal percentile value (threshold). Low values characterize the first group, while high values characterize the second group.

**Fig. 4:**
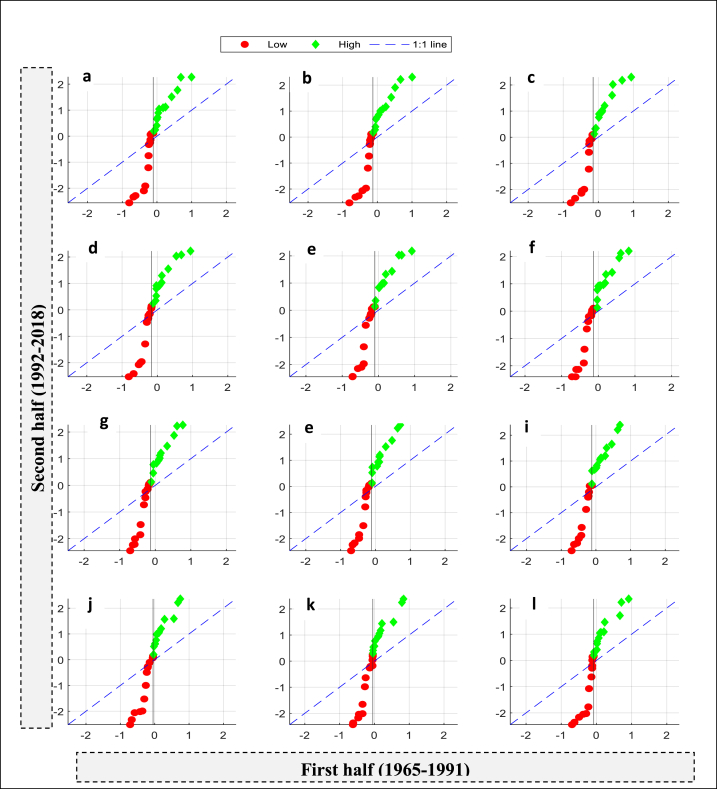
MSSI trends detected by innovative trend analysis method during January to December at a station S1. (a) January, (b) February, (c) March,(d) April (e) May, (f) June, (g) July, (h) August, (i) September, (j) October,(k) November, and (l) December.

**Fig. 5:**
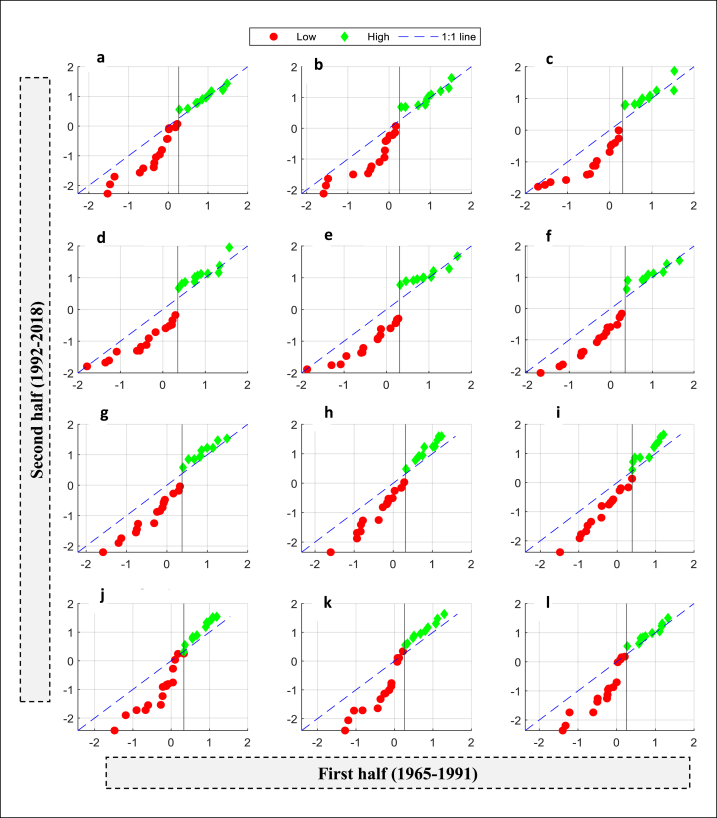
MSSI trends detected by innovative trend analysis method during January to December at a station S2. (a) January, (b) February, (c) March,(d) April (e) May, (f) June, (g) July, (h) August, (i) September, (j) October,(k) November, and (l) December.

The basis for selecting the optimal threshold is to effectively separate higher and lower drought values, facilitating the analysis of drought patterns and trends for each category. By establishing a clear separation between the two categories, any overlap or mixing of hydrological drought data is avoided, enabling a focused examination of each group's distinct trends. Also, this separation allows for more effective identification and understanding of the patterns associated with low and high drought values. While determining the specific threshold value can be complex, this study employed a trial-and-error method to compute the optimal threshold value for each station that effectively separates the higher drought data from the lower data. Thus, this procedure would help to conduct a comprehensive analysis of drought patterns and trends. To achieve this, the 60th percentile is used as the threshold for low values for the S1 station, while the remaining 40th percentile (100-60) is used as the threshold for high values (Fig. 4(a to l)). Similarly, the 35th percentile is used as the threshold for low values for the S2 station, while the 65th percentile (100-35) is used as the threshold for high values (Fig. 5(a to l)). Each station has its own threshold value, which differs from the threshold value of other station. This difference in threshold values is attributed to the variations in drought characteristics observed between the stations. The graphs presented clearly demonstrate that the S2 stations have experienced a significant decrease in low drought values for all months. However, the increasing trend in high drought values is only marginal. The drought patterns for the second station (S1) are quite complex. While a decreasing trend was observed in low drought values, an increasing pattern was detected for higher drought values in all months of the year. Table 3, Table 4 show the station-wise trend indicator, the slope of the trend indicator, the upper confidence level (Up), and the lower confidence level (Lo) of ITAM for the monthly MSSI of S1 and S2 stations. The trend analysis results for both stations revealed that the months of October, November, and December exhibit the highest slope (i.e., $S_{ITAM})$ in terms of low values compared to the other months. This implies that the severity of drought during these months is remarkably high.

**Table 3:** The results of innovative trend test of MSSI for S1 station.

Month	Lower			High		
	Slope	Up*	Lo**	Slope	Up*	Lo**
Jan	0.1235	0.0058	−0.0058	−0.0373	0.0017	−0.0017
Feb	0.1225	0.0053	−0.0053	−0.0390	0.0016	−0.0016
Mar	0.1175	0.0048	−0.0048	−0.0402	0.0016	−0.0016
Apr	0.1135	0.0040	−0.0040	−0.0410	0.0019	−0.0019
May	0.1162	0.0038	−0.0038	−0.0396	0.0017	−0.0017
Jun	0.1172	0.0032	−0.0032	−0.0400	0.0016	−0.0016
Jul	0.1169	0.0025	−0.0025	−0.0419	0.0011	−0.0011
Aug	0.1179	0.0031	−0.0031	−0.0429	0.0011	−0.0011
Sep	0.1238	0.0024	−0.0024	−0.0415	0.0011	−0.0011
Oct	0.1293	0.0046	−0.0046	−0.0384	0.0015	−0.0015
Nov	0.1369	0.0032	−0.0032	−0.0367	0.0018	−0.0018
Dec	0.1437	0.0055	−0.0055	−0.0368	0.0018	−0.0018

The symbols *, and ** are the upper and lower critical bounds of the trend stope at 5%significance level.

**Table 4:** The results of innovative trend test of MSSI for S2 station.

Lower				High		
Month	Slope	Up*	Lo**	Slope	Up*	Lo*
Jan	0.0339	0.0047	−0.0047	−0.0027	0.0028	−0.0028
Feb	0.0322	0.0055	−0.0055	−0.0057	0.0057	−0.0057
Mar	0.0330	0.0053	−0.0053	−0.0163	0.0072	−0.0072
Apr	0.0336	0.0037	−0.0037	−0.0181	0.0061	−0.0061
May	0.0371	0.0037	−0.0037	−0.0138	0.0052	−0.0052
Jun	0.0381	0.0025	−0.0025	−0.0125	0.0045	−0.0045
Jul	0.0372	0.0024	−0.0024	−0.0171	0.0032	−0.0032
Aug	0.0357	0.0022	−0.0022	−0.0250	0.0024	−0.0024
Sep	0.0370	0.0015	−0.0015	−0.0255	0.0036	−0.0036
Oct	0.0425	0.0044	−0.0044	−0.0256	0.0017	−0.0017
Nov	0.0409	0.0042	−0.0042	−0.0267	0.0018	−0.0018
Dec	0.0393	0.0045	−0.0045	−0.0102	0.0032	−0.0032

The symbols *, and ** are the upper and lower critical bounds of the trend stope at 5%significance level.

### 4.2 Modeling results

**Fig. 6:**
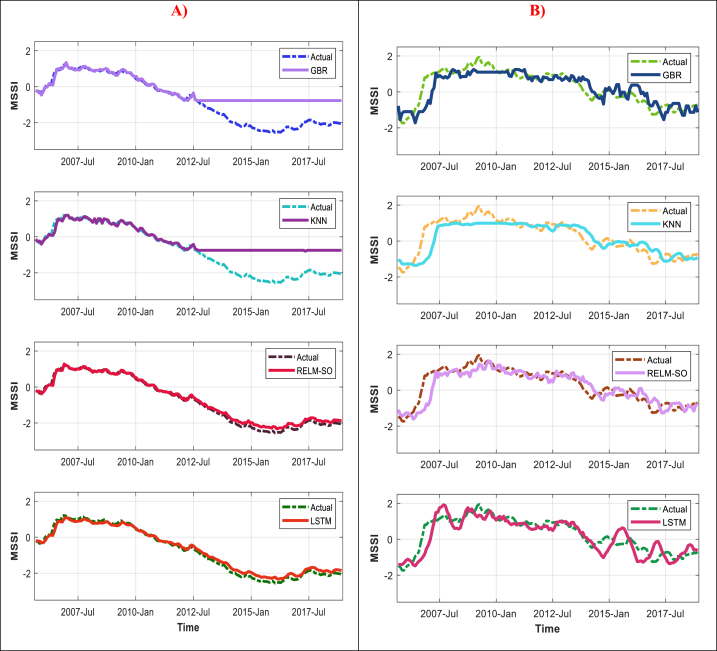
Line graph showing the calculated and forecasted hydrological drought by the applied models for both stations. A) S1station. B) S2 station.

The current research utilized four distinct machine learning models to forecast the hydrological drought at two locations in Malaysia, with a forecast horizon of up to six months. The capability of the hybrid forecasting model (RELM-SO) and LSTM model as deep learning algorithms to forecast hydrological drought for two stations in Malaysia up to six months in advance is discussed in this section of the study. Additionally, two standalone ML-based models (e.g., GBR and KNN) were used as a benchmark to assess the efficiency of RELM-SO and LSTM. The quantitative assessment results of the forecasting models throughout the training and testing phases for the S1 and S2 stations are provided in Table 5, Table 6. Overall, all models perform satisfactorily in the training stage, with the GBR model exhibiting clear superiority. Notably, during the training stage, a model was given input and output data, whereas only input data was provided in the testing phase. Accordingly, the latter stage is more precise in evaluating the effectiveness of the model's performance [77]. By analyzing the results presented in Table 5, Table 6, it is apparent that the performance of GBR is notably different between the training and testing stages, unlike the other models (e.g., LSTM and RELM-SO) that exhibited excellent performance. Besides, the RELM-SO has illustrated the best accuracy in forecasting the MSSI from one to six months ahead, followed by LSTM, GBR, and KNN. As an illustration, the RELM-SO model demonstrates remarkable accuracy in forecasting a one-month drought in advance (MSSI_t+1_) for the S1 station during the testing phase. This model yields lower values of *RMSE* (0.1453) and *MAE* (0.1164), as well as higher values of *NSE* (0.9012) and *WI* (0.9966). Nonetheless, the LSTM model also shows good accuracy, lower than the RELM-SO model, with an *RMSE* of 0.1677, *MAE* of 0.1416, *NSE* of 0.8799, and *WI* of 0.9954. Similarly, for station S2, the hybrid model's performance was more effected than comparable models, reporting fewer forecasting error (*RMSE* = 0.1211, and *MAE* = 0.0909), and higher accuracy (*NSE* = 0.8941, and *WI* = 0.9960), followed by LSTM (*RMSE* = 0.1383, *MAE* = 0.1060, *NSE* = 0.8765, and *WI* = 0.9947). Generally, there was an outstanding variation in the capacity of the model in forecasting drought for the S1 and S2 stations, likely due to differences in their respective training data quality. Specifically, the absence of severe drought conditions in the training data for station S1 resulted in a relatively higher forecasting error during the testing phase. To further illustrate this point, a line graph (Fig. 6(A, and B)) was created to depict how the models simulated the changes in drought values over time. The results indicate that classical models such as GBR and KNN could not accurately simulate the most critical drought events, particularly in the S1 station. On the other hand, advanced models like LSTM and RELM-SO provided highly precise results in simulating these critical events, even when they were not present in the training data. This suggests that these models have excellent generalization capabilities. Overall, the RELM-SO has simulated the MSSI for both stations much better than LSTM, reflecting its capability to forecast the hydrological drought.

**Table 5:** Result AI models for S1 station.

Lead Time	Models	Training Phase				Testing Phase			
		MAE	RMSE	NSE	WI	MAE	RMSE	NSE	WI
MSSI_t+1_	GBR	0.0318	0.0406	0.9403	0.9992	0.6181	0.8977	0.4755	0.8114
	KNN	0.0651	0.0856	0.8778	0.9964	0.6370	0.9144	0.4595	0.8015
	RELM-SO	0.0513	0.0661	0.9037	0.9978	0.1164	0.1453	0.9012	0.9966
	LSTM	0.0809	0.1083	0.8482	0.9936	0.1416	0.1677	0.8799	0.9954
MSSI_t+2_	GBR	0.0519	0.0668	0.9026	0.9978	0.6750	0.9394	0.4293	0.7950
	KNN	0.1756	0.2768	0.6705	0.9467	0.8575	1.1621	0.2750	0.6983
	RELM-SO	0.0853	0.1066	0.8400	0.9943	0.2445	0.3044	0.7933	0.9840
	LSTM	0.1346	0.1604	0.7475	0.9879	0.3051	0.3450	0.7420	0.9807
MSSI_t+3_	GBR	0.0400	0.0504	0.9250	0.9987	0.7312	0.9869	0.3818	0.7775
	KNN	0.1485	0.1968	0.7217	0.9772	0.8142	1.1021	0.3116	0.7286
	RELM-SO	0.1147	0.1409	0.7850	0.9900	0.3233	0.4000	0.7267	0.9710
	LSTM	0.1135	0.1415	0.7874	0.9898	0.4180	0.4941	0.6466	0.9548
MSSI_t+4_	GBR	0.0375	0.0481	0.9298	0.9989	0.8084	1.0658	0.3165	0.7456
	KNN	0.2499	0.3817	0.5318	0.8802	0.9598	1.2368	0.1884	0.6471
	RELM-SO	0.1338	0.1670	0.7495	0.9858	0.4990	0.6363	0.5780	0.9168
	LSTM	0.1099	0.1384	0.7941	0.9904	0.6270	0.6904	0.4699	0.9168
MSSI_t+5_	GBR	0.1978	0.2532	0.6294	0.9622	0.7972	1.0271	0.3260	0.7221
	KNN	0.2816	0.4322	0.4725	0.8328	1.0022	1.2676	0.1526	0.6236
	RELM-SO	0.1521	0.1920	0.7151	0.9809	0.5098	0.6338	0.5689	0.9176
	LSTM	0.1456	0.1788	0.7272	0.9834	0.6169	0.7337	0.4784	0.8886
MSSI_t+6_	GBR	0.1334	0.1686	0.7499	0.9853	0.8559	1.0875	0.2789	0.7274
	KNN	0.2777	0.4196	0.4793	0.8468	1.0100	1.2720	0.1490	0.6258
	RELM-SO	0.1695	0.2122	0.6821	0.9765	0.5350	0.6547	0.5492	0.9119
	LSTM	0.1477	0.1811	0.7230	0.9832	0.6278	0.7311	0.4711	0.8947

**Table 6:** Result AI models for S2 station.

Lead Time	Models	Training Phase				Testing Phase			
		MAE	RMSE	NSE	WI	MAE	RMSE	NSE	WI
MSSI_t+1_	GBR	0.1046	0.1380	0.8730	0.9950	0.1140	0.1502	0.8672	0.9936
	KNN	0.1441	0.1918	0.8251	0.9900	0.1342	0.1820	0.8437	0.9906
	RELM-SO	0.1040	0.1377	0.8738	0.9951	0.0909	0.1211	0.8941	0.9960
	LSTM	0.1048	0.1423	0.8728	0.9947	0.1060	0.1383	0.8765	0.9947
MSSI_t+2_	GBR	0.0783	0.1023	0.9053	0.9973	0.2023	0.2587	0.7627	0.9807
	KNN	0.2414	0.3070	0.7078	0.9717	0.2171	0.2968	0.7454	0.9727
	RELM-SO	0.1782	0.2292	0.7843	0.9861	0.1590	0.2125	0.8135	0.9872
	LSTM	0.1804	0.2315	0.7818	0.9858	0.1843	0.2404	0.7839	0.9838
MSSI_t+3_	GBR	0.1563	0.1987	0.8112	0.9895	0.2570	0.3337	0.6985	0.9679
	KNN	0.2813	0.3528	0.6601	0.9626	0.2567	0.3523	0.6990	0.9617
	RELM-SO	0.2405	0.3051	0.7095	0.9746	0.1590	0.2125	0.8135	0.9872
	LSTM	0.2315	0.2956	0.7203	0.9759	0.2415	0.3159	0.7168	0.9713
MSSI_t+4_	GBR	0.1283	0.1671	0.8453	0.9926	0.3153	0.4138	0.6302	0.9517
	KNN	0.3587	0.4451	0.5674	0.9312	0.3253	0.4306	0.6185	0.9355
	RELM-SO	0.2881	0.3631	0.6525	0.9629	0.2641	0.3540	0.6902	0.9633
	LSTM	0.3007	0.3684	0.6373	0.9628	0.2934	0.3907	0.6559	0.9559
MSSI_t+5_	GBR	0.2016	0.2624	0.7571	0.9812	0.3594	0.4559	0.5786	0.9383
	KNN	0.3856	0.4789	0.5356	0.9203	0.3521	0.4684	0.5871	0.9242
	RELM-SO	0.3264	0.4147	0.6068	0.9502	0.3004	0.4060	0.6477	0.9508
	LSTM	0.2900	0.3591	0.6506	0.9639	0.3436	0.4258	0.5970	0.9482
MSSI_t+6_	GBR	0.2858	0.3662	0.6567	0.9611	0.3629	0.4833	0.5716	0.9250
	KNN	0.3885	0.4930	0.5334	0.9206	0.3649	0.5023	0.5693	0.9164
	RELM-SO	0.3613	0.4580	0.5660	0.9371	0.3373	0.4570	0.6018	0.9353
	LSTM	0.2034	0.2541	0.7556	0.9831	0.3696	0.4751	0.5637	0.9353

**Fig. 7:**
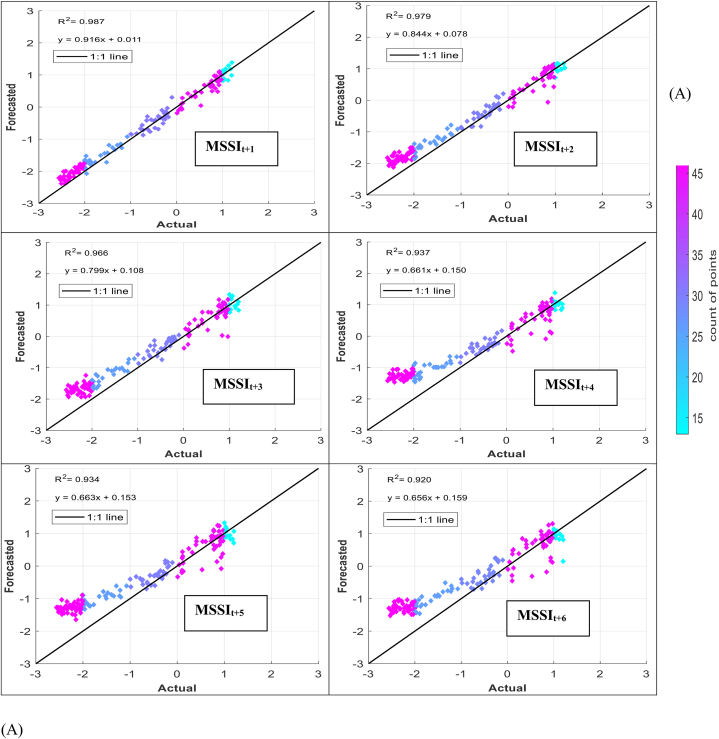

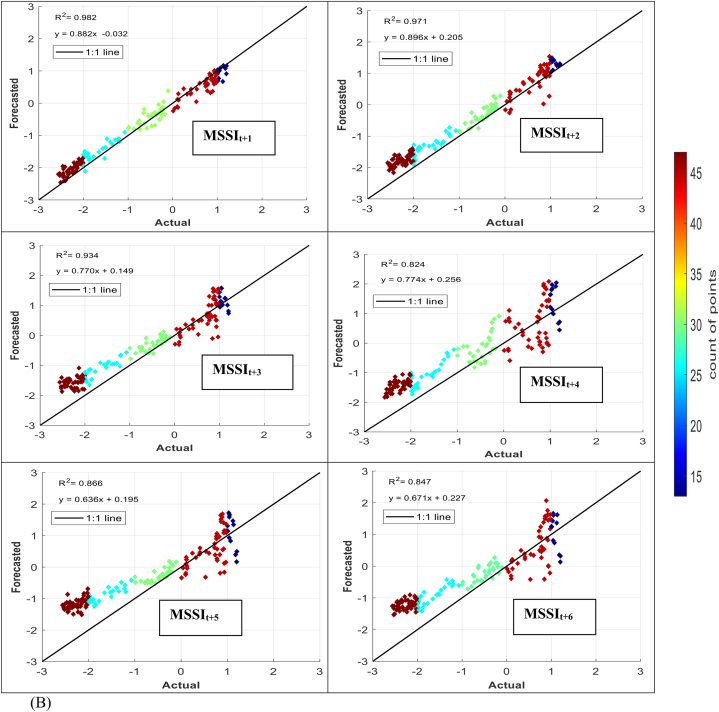

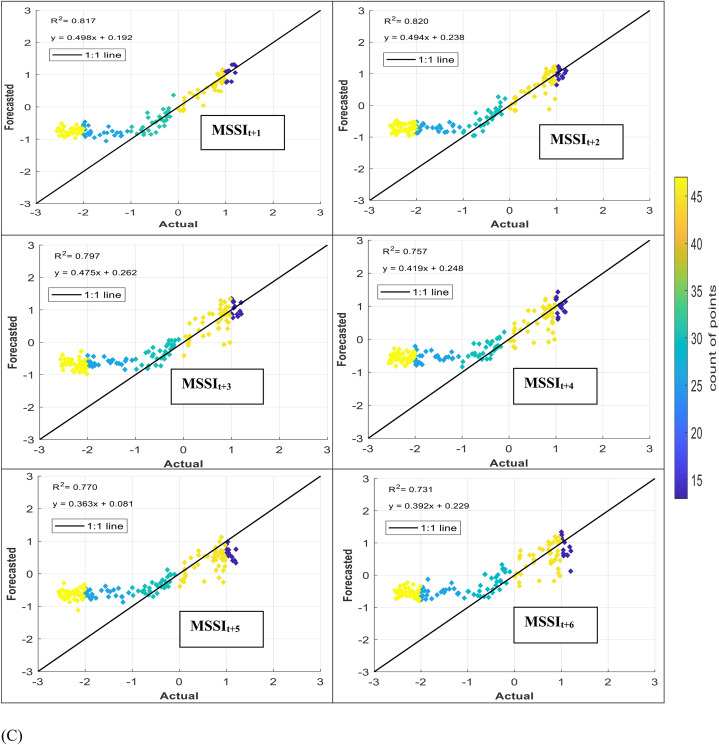

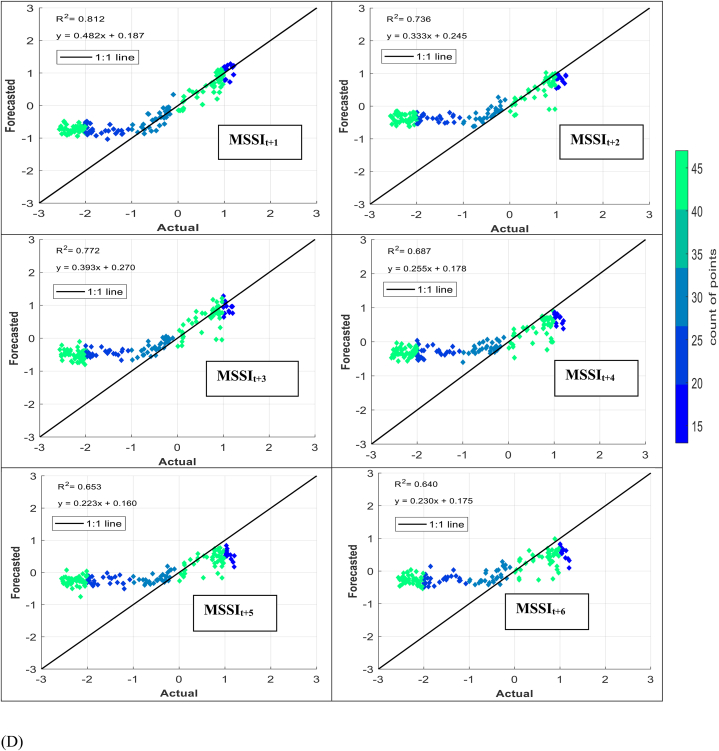

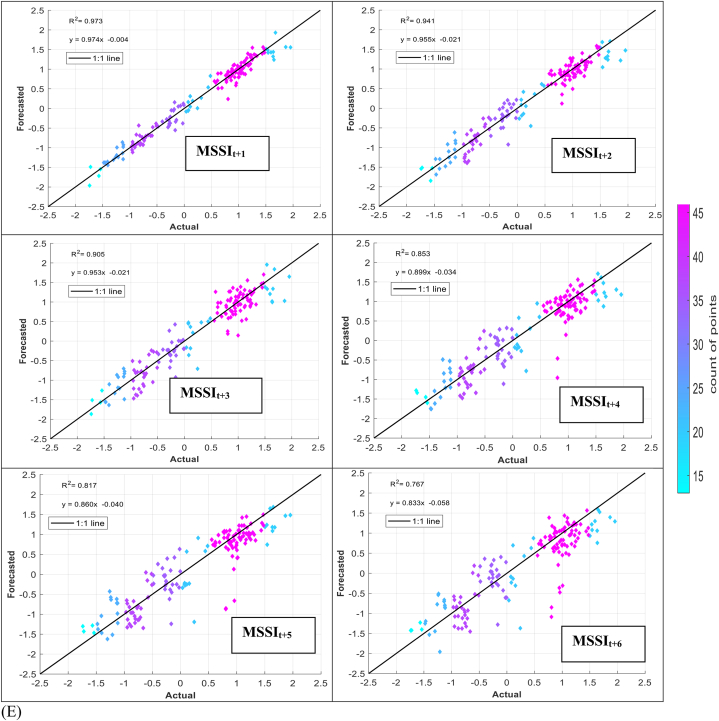

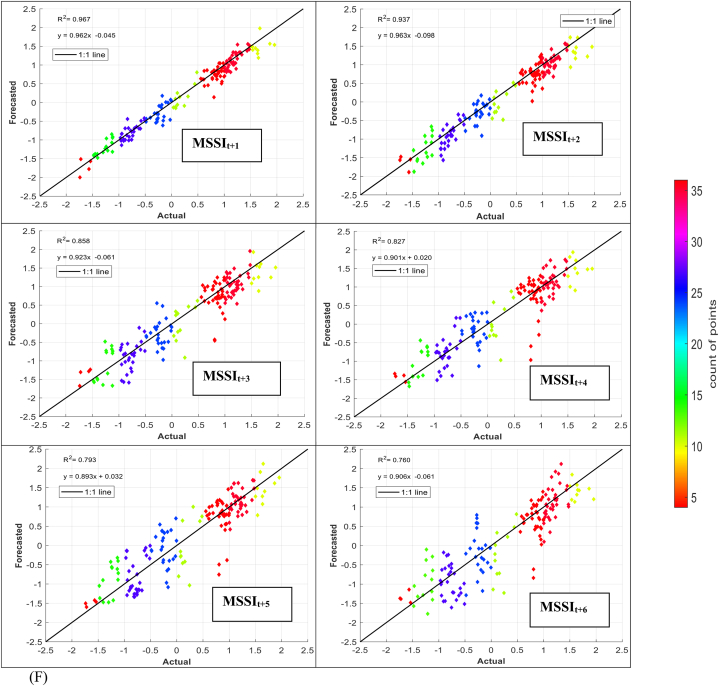

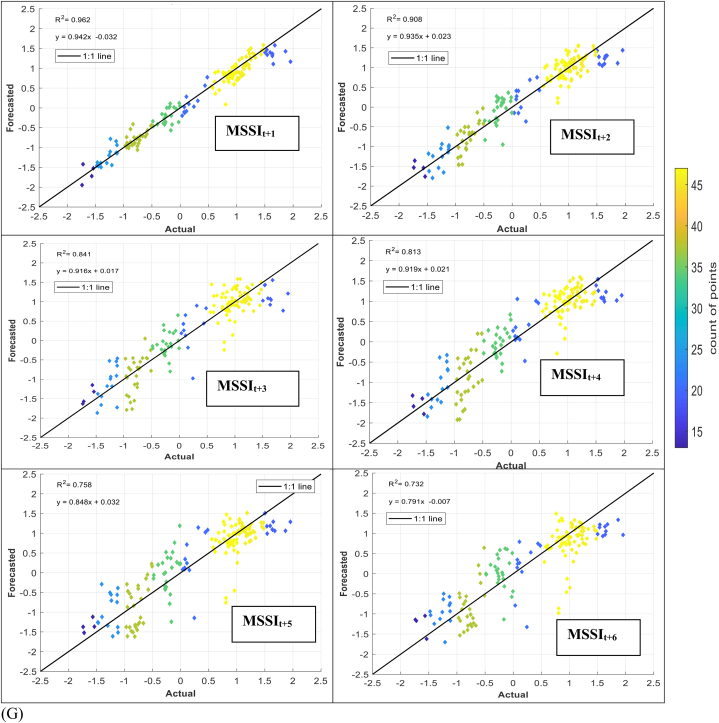

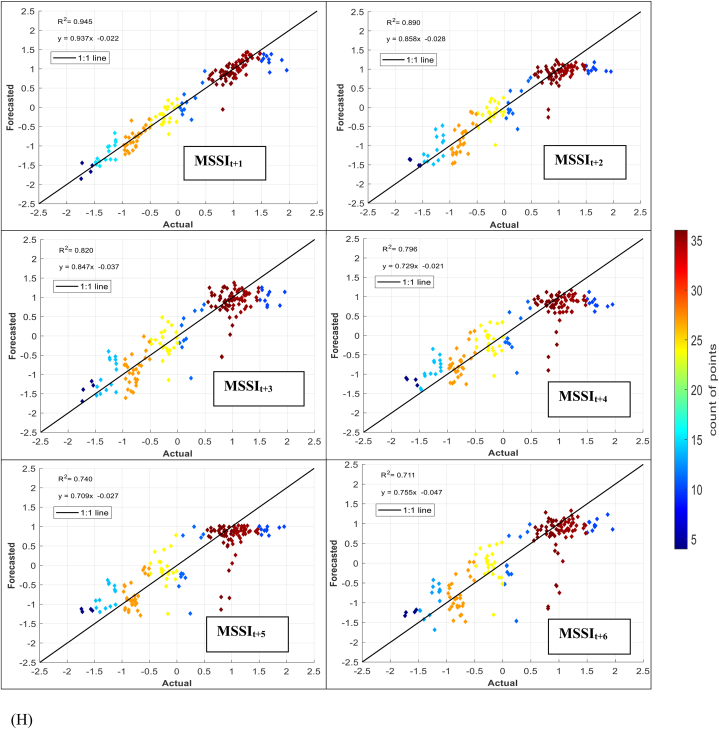
Comparison between calculated MSSI and forecasted values for S1 station through the testing phase: (A) RELM-SO, (B) LSTM, (C) GBR, (D) KNN models. Comparison between calculated MSSI and forecasted values for S2 station through the testing phase: (E) RELM-SO, (F) LSTM, (G) GBR, (H) KNN models.

The models' performance in simulating observed MSSI at both stations several months ahead (from one to six) was evaluated using scatter plots for testing phases (Fig. 7 (A-H)). These plots provide informative visualizations of the deviation between forecasted and calculated MSSI and the correlation of determination (*R*^*2*^) between them. The density distribution of real drought data was also calculated, and the colored points indicate the corresponding projected values. The forecasted results illustrated that the RELM-SO model outperforms the other models regarding higher *R*^*2*^ values. The *R*^*2*^ ranges for several models at S1 and S2 stations were determined. At the S1 station, the RELM-SO model had the highest *R*^*2*^ range of 0.987 to 0.920, with LSTM following closely behind at 0.982 to 0.842. GBR and KNN had lower ranges of 0.817 to 0.731 and 0.812 to 0.640, respectively. Similarly, for the S2 station, the RELM-SO model had the highest *R*^*2*^ values (0.973–0.767), followed by LSTM with a range of 0.967 to 0.760. GBR and KNN had lower ranges of 0.962 to 0.732 and 0.945 to 0.711, respectively.

#### 4.2.1 Analysis of forecasting models based on turning point detection

**Fig. 8:**
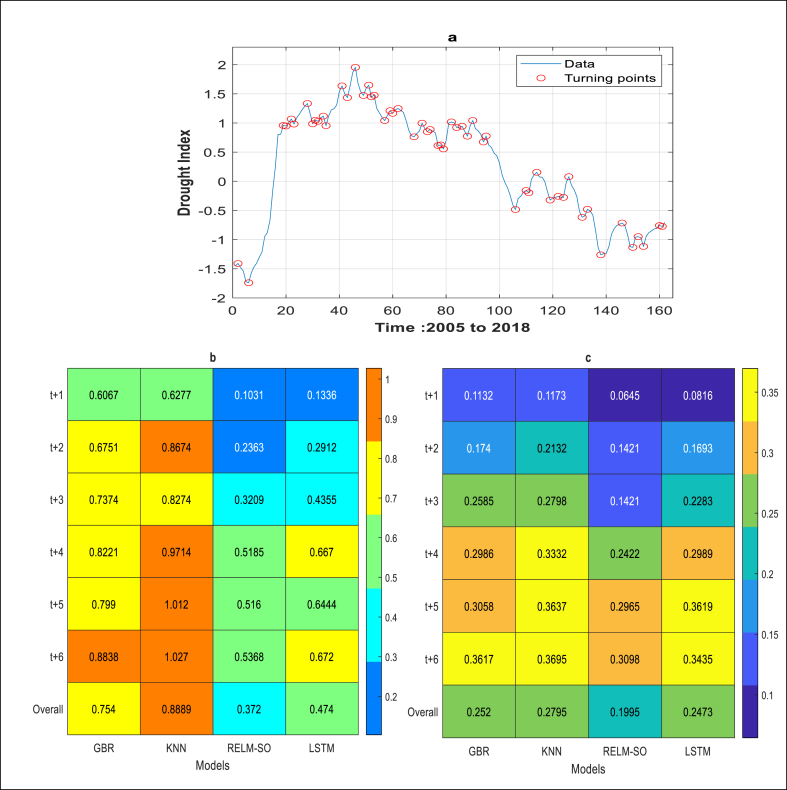
Turning point detection and analysis: a) illustration of the algorithm's capability in detecting critical and turning points in drought time series data. b) *AAETP* results for station S1.c) *AAETP* results for station S2.

The analysis of the model's capability to capture drought turning points has been assessed. Fig. 8a demonstrates how the applied algorithm identifies the critical and turning points in the calculated drought data. Based on the results offered in Fig. 8 (b, and c), the hybrid model (RELM-SO) demonstrated more accurate capturing of turning points compared to LSTM and other ML-based models, as evidenced by its lowest value of *AAETP*. The superiority of RELM-SO was quantified in terms of its ability to reduce the *AAETP* indicator. The findings indicated a significant improvement in forecasting accuracy of 50.64 %, 58.16 %, and 21.52 % using RELM-SO compared to GBR, KNN, and LSTM models, respectively, for the S1 station. Similarly, for the S2 station, the results demonstrated a significant enhancement in forecasting accuracy of 20.82 %, 28.61 %, and 19.32 % when compared to GBR, KNN, and LSTM models, respectively.

**Fig. 9:**
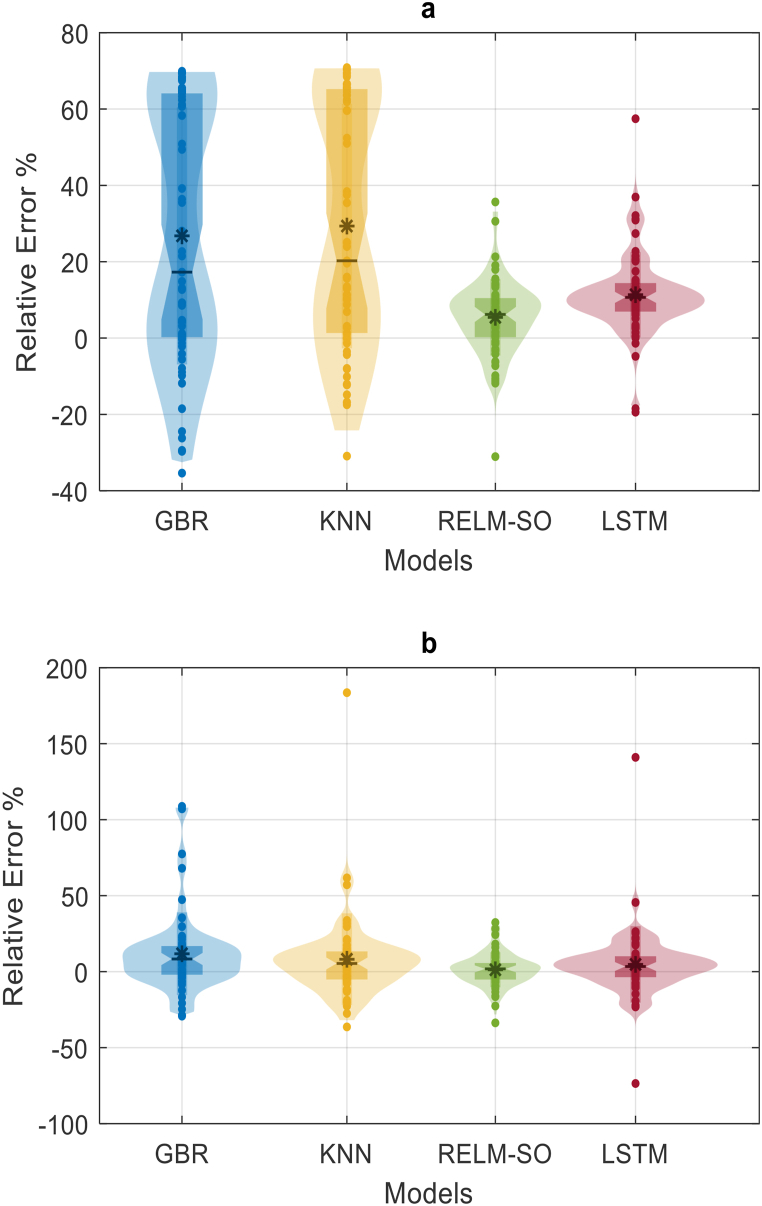
Depicts violin diagrams illustrating the relative error percentages. Panel a) represents the S1 station, while panel b) corresponds to the S2 station.

An analysis of the relative errors in forecasting tuning and critical points is conducted to discern the optimal forecasting model. The best model is characterized by its accuracy in pinpointing these points. Violin plots are used to present the relative error percentages (Fig. 9(a and b)) due to their capacity to comprehensively display data elements like mean, median, IQR, and distribution. For station S1, it's evident that the KNN and GBR models yield high errors, oscillating between −33 % and 69 %, in forecasting the turning points for one month ahead, as demonstrated in Fig. 9a. Meanwhile, the LSTM model yields a smaller error range, fluctuating between −3% and −40 %; only two points that account for a mere 3.13 % of total points surpass this range. Besides this, the RELM-SO model registers the narrowest error range, hovering around −18 % to −20 %, only three points slightly exceeding this span. It's clear that the RELM-SO is the most reliable model for forecasting drought at station S1. Similarly, for station S2, the hybrid model manifests the least relative errors, as shown in Fig. 9b, affirming its superiority over the LSTM and all other models.

#### 4.2.2 Reliability analyses

The study suggests that further statistical assessments are necessary to determine whether the adopted model (RELM-SO) has better accuracy for long-term drought forecasting than other models. Some researchers propose using reliability assessment (*RA*), which is an advanced statistical indicator commonly used in the literature to evaluate model performance and consistency [88]. *RA* can also determine whether the forecasting models meet the minimum requirement for acceptable accuracy [73]. Thus, *RA* metric is a crucial tool for determining the effectiveness of forecasting models. *RA* can be mathematically expressed using Equation (34).

(34) $$\left(RA\right)=\left(\frac{100\%}{Ns}\right)\sum_{k=1}^{Ns}j_{k}$$

where Ns is the total number of MSSI $j_{i}$ is an *RA* index calculated by two steps. The term $j_{k}$ is calculated via two steps. First, it is crucial to determine the Relative Absolute Error (*RAE*) as a vector of $j_{k}$ component using Equation (35).

(35) $${RAE}_{k}=\left|\frac{{MSWI}_{k\left(Observed\right)}-{MSWI}_{k\left(Forecasted\right)}\;}{{MSWI}_{k\left(Observed\right)}}\right|$$

In the second step, the equivalent value of $j_{k}$ is determined based on the threshold (φ) of an adequate drought parameter and the ${RAE}_{k}$. If the ${RAE}_{k}$ is greater than α, then $j_{k}$ is assigned a value of zero. However, if ${RAE}_{k}$ is less than or equal to φ, then $j_{k}$ is assigned a value of one. The Chinese Standards recommend an optimal value of 0.2 (20 %) for φ.

**Fig. 10:**
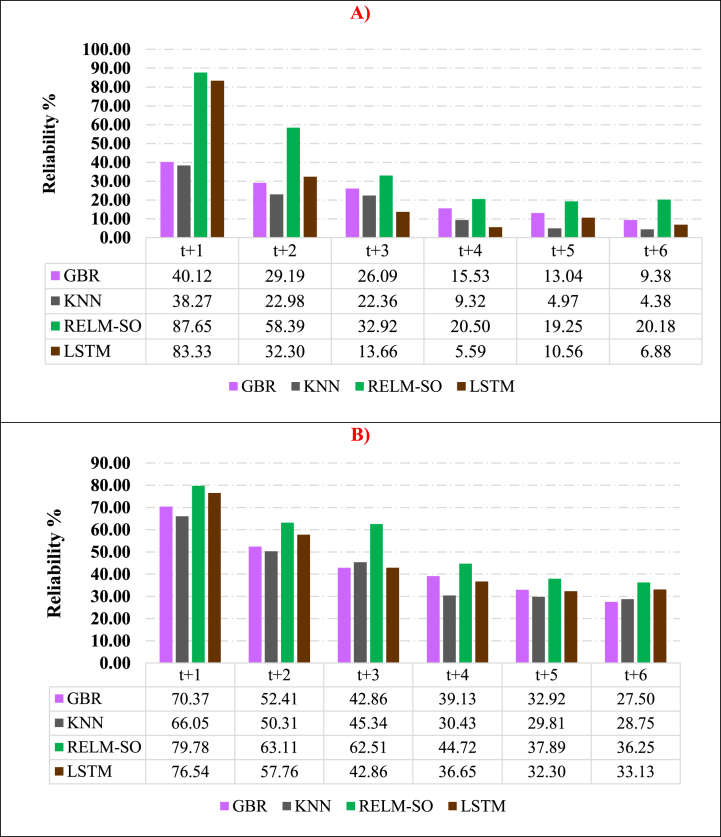
Results of reliability analysis for assessing model efficiencies in forecasting MSSI for one to six months in advance. A) S1 station, and B) S2 station.

Based on the reliability metric, it is evident from Fig. 10 (A, and B) that the RELM-SO model outperforms other models in terms of dependability. The results demonstrate that, when forecasting MSSI several months ahead (*t*+1 to *t*+6), the proposed model outperforms other comparable models, as evidenced by the highest *RA* ranging from 87.65 % to 20.18 % for the S1 station (see Fig. 10 A) and from 79.78 % to 36.25 % for the S2 station (see Fig. 10 B). Thus, the study confirms the significant superiority of the proposed model. As forecasting accuracy declines with increasing drought forecast lead time, conducting a *RA* can provide valuable insights into identifying the optimal number of months in advance for a model to produce high-precision forecasts. Based on illustrated results in Fig. 10(A, and B), it can be inferred that the RELM-SO can produce reliable forecasts for the first station up to two months in advance, while for the second station, the forecast horizon is limited to three months.

#### 4.2.3 Discussion

Multi-month ahead drought forecasting is imperative in establishing strategies to mitigate the impacts of drought, manage water resources efficiently, and develop an early warning system. This study employed multiple models to predict one to six-month-ahead droughts for two particular dry regions in Malaysia. The RELM-SO model proved to be more accurate than the LSTM model, benchmark models like KNN, GBR, and others that were developed in earlier works. Quantitative results indicate that the RELM-SO model performs better in multi-month drought forecasting. This can be attributed to the model's structure, which amalgamates the advantages of two robust algorithms: RELM and SO. Significantly, SO was utilized to train the RELM due to its proven effectiveness in solving a variety of real-world engineering problems and surpassing other traditional and new algorithms (e.g., Coyote Optimization Algorithm, Moth-flame Optimization, Harris Hawks Optimizer, Whale Optimization Algorithm, etc.) [83]. A noteworthy element is the RELM's regularization parameter, which enhances the models' ability to yield more accurate forecasts, particularly with testing data [82]. This parameter also augments the model's capability by striking a balance between two forms of risk: structural risk (related to the models' coefficients) and empirical risk (related to forecasting errors).

Further analysis is necessary to ensure the superiority of the suggested RELM-SO model in forecasting drought one month in advance in Malaysia. This involves evaluating the accuracy of the RELM-SO model by comparing it with other models established in previous studies. One of the studies used advanced models based on a combination of wavelet transform (WA) with ANFIS, creating the WANFIS model [89]. Similarly, the second model was established based on a combination of WA, ARIMA, and ANN, creating the WAANN model. Quantitative analysis showed that the latter model had higher forecasting accuracy for drought, with an *R*^*2*^ value of 0.9603. Another study investigated the capacity of a model established through a combination of WA and ANN, which achieved a higher result with an *R*^*2*^ of 0.938 [90]. Moreover, other researchers presented advanced models using Fuzzy-SVR, Boosted-SVR, and classical SVR to forecast drought and found that the Fuzzy-SVR model performed best with an *R*^*2*^ value of 0.903 [91]. Additionally, literature has investigated single models such as ANN and found that it can provide a good value of *R*^*2*^, reaching 0.833 [92]. Finally, an advanced combining model was developed based on the integration of WA, ARIMA, and ANN, which provided higher results with an *R*^*2*^ of 0.872 [92]. Furthermore, some researchers have used a novel model based on ANN coupled with the Firefly Algorithm to forecast drought at various temporal scales using the SPI index [93]. The best results obtained in these studies yielded an *R*^*2*^ value of 0.887. Overall, the reviewed models provided satisfactory accuracy, with *R*^*2*^ values ranging from 0.9603 to 0.833. However, the accuracy forecasting of these models is still less than that obtained in the RELM-SO model, which provides a higher forecasting accuracy ranging from 0.987 to 0.973.

The poor forecasting results of the GBR and KNN models for the S1 station from 2012 to 2018, specifically for peak drought values, suggest that these models may not effectively capture the complex patterns and dynamics of hydrological drought data. This indicates that they might oversimplify the relationships between the input features and the target variable, making them unsuitable for accurately forecasting drought values. Moreover, the relatively high correlations between the features and outputs negatively affect ensemble models like GBR, leading to poor forecasting results [94].

The analysis of turning points using the *AAETP* indicator reveals that the RELM-SO model performs the best, followed by LSTM. The Violin diagram presentation provides valuable information on the quality forecasting of these models. Observing Fig. 9, it is evident that the distribution of relative error for RELM-SO closely resembles a normal distribution, unlike other models, in both studied stations. Notably, all models, except RELM-SO, exhibit noticeable skewness towards positive and negative values in both stations. Furthermore, in station SI, the relative error distribution of the LSTM model is less accurate compared to its competitor, RELM-SO. The LSTM model tends to provide underestimating forecasts, with most relative error records above zero. Consequently, based on the lowest forecasting error at the turning points for both stations and the normality of their distribution, it is confirmed that RELM-BWO is the superior model.

In the studied area, the achieved results may have a notable impact on water resource management, agriculture, and disaster preparedness. Accurately anticipating drought conditions and utilizing advanced drought trend analysis may provide stakeholders and decision-makers with significant information on drought dynamics. This enables them to implement ecosystem protection measures, sustainable water resource management strategies, and advanced drought warning systems. Such a comprehensive and precise analysis may effectively mitigate the environmental impacts of drought.

## 5 Conclusion

Accurate trend analysis and reliable drought forecasting are both crucial for understanding drought variability, informing stakeholders about consequences, and facilitating effective water resource management to mitigate the negative impacts of drought. The hydrological drought at two hydrological stations in West Malaysia was computed using MSSI based on streamflow data spanning 58 years (1961–2018). This study compares the performance of four models - the hybrid model (RELM-SO), deep learning model (LSTM), GBR, and KNN - in forecasting droughts over a forecasting horizon of one to six months ahead. The results highlight that the hybrid model outperforms the comparable models and several novel and recent models developed in the literature. Considering the presence of significant autocorrelation in the drought time series data, the models were evaluated based on their capacity to capture critical and turning drought points. The RELM-SO demonstrates superior performance in capturing critical and turning points, as *AAETP* values indicate. The hybrid model also exhibits substantial improvements in forecast accuracy compared to GBR, KNN, and LSTM models, with reductions in *AAETP* ranging from 21.52 % to 58.16 % for the S1 station and improvements of 19.32 %–28.61 % for the S2 station. Furthermore, the analysis reveals that RELM-SO can provide reliable forecasts up to two months in advance for the S1 station, while the forecast horizon for the S2 station is limited to three months in advance. These findings highlight the effectiveness of the hybrid model in drought forecasting, surpassing other models in both capturing turning points and improving forecast accuracy.

Additionally, this study addresses the need for comprehensive drought trend analysis using the ITAM method. Trend analysis revealed that the S2 station experienced a significant decrease in low drought values for all months, while the increasing trend in high drought values was marginal. The drought patterns for the S1 station were complex, with a remarkable decreasing trend in low drought values but an increasing trend in higher drought values throughout the year has been observed. Besides, the months of October, November, and December exhibited the highest slope (i.e., $S_{ITAM}$), showing a higher frequency of drought events than in other months. Overall, this study showcases the effectiveness of the hybrid model (RELM-SO) in accurately forecasting droughts, capturing critical turning points, and providing valuable insights through effective drought trend analysis, thereby enhancing water resource management and decision-making.

## Data availability

The supporting data for this study are owned by the Department of Irrigation and Drainage Malaysia. Due to restrictions, the authors are not authorized to share the data publicly.

## CRediT authorship contribution statement

**Mohammed Majeed Hameed:** Writing - original draft, Visualization, Software, Methodology, Conceptualization. **Siti Fatin Mohd Razali:** Validation, Supervision, Project administration, Data curation, Conceptualization. **Wan Hanna Melini Wan Mohtar:** Validation, Investigation, Formal analysis, Data curation. **Majed Omar Ahmad Alsaydalani:** Visualization, Funding acquisition, Data curation. **Zaher Mundher Yaseen:** Writing - review & editing, Validation, Investigation.

## Declaration of competing interest

The authors declare that they have no known competing financial interests or personal relationships that could have appeared to influence the work reported in this paper.
