# Supplementary material for: Deep learning versus hybrid regularized extreme learning machine for multi-month drought forecasting: A comparative study and trend analysis in tropical region
Source: Heliyon. 2023 Nov 28;10(1):e22942. doi: 10.1016/j.heliyon.2023.e22942 (PMC10767141; doi:10.1016/j.heliyon.2023.e22942)
Supplement: Multimedia component 1 [file mmc1.docx]

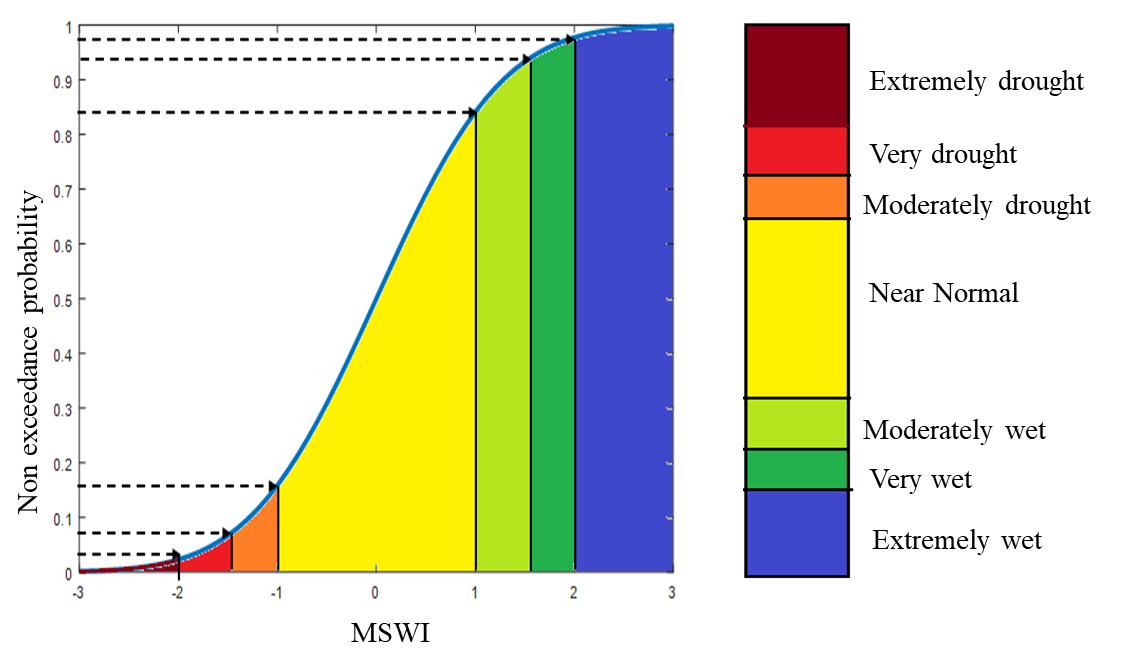


MSSI value

Appendix A: Drought classes based on the MSSI value.

No trend

Straight line (1:1)

Increasing Trend Zone

Decreasing Trend Zone

Second half of the time series, Y_j_

First half of the time series, Xi

Low

High

Appendix B: Illustration of Innovative Trend Analysis Method for identifying trends (increasing, decreasing, or no Trend) in drought data.

| 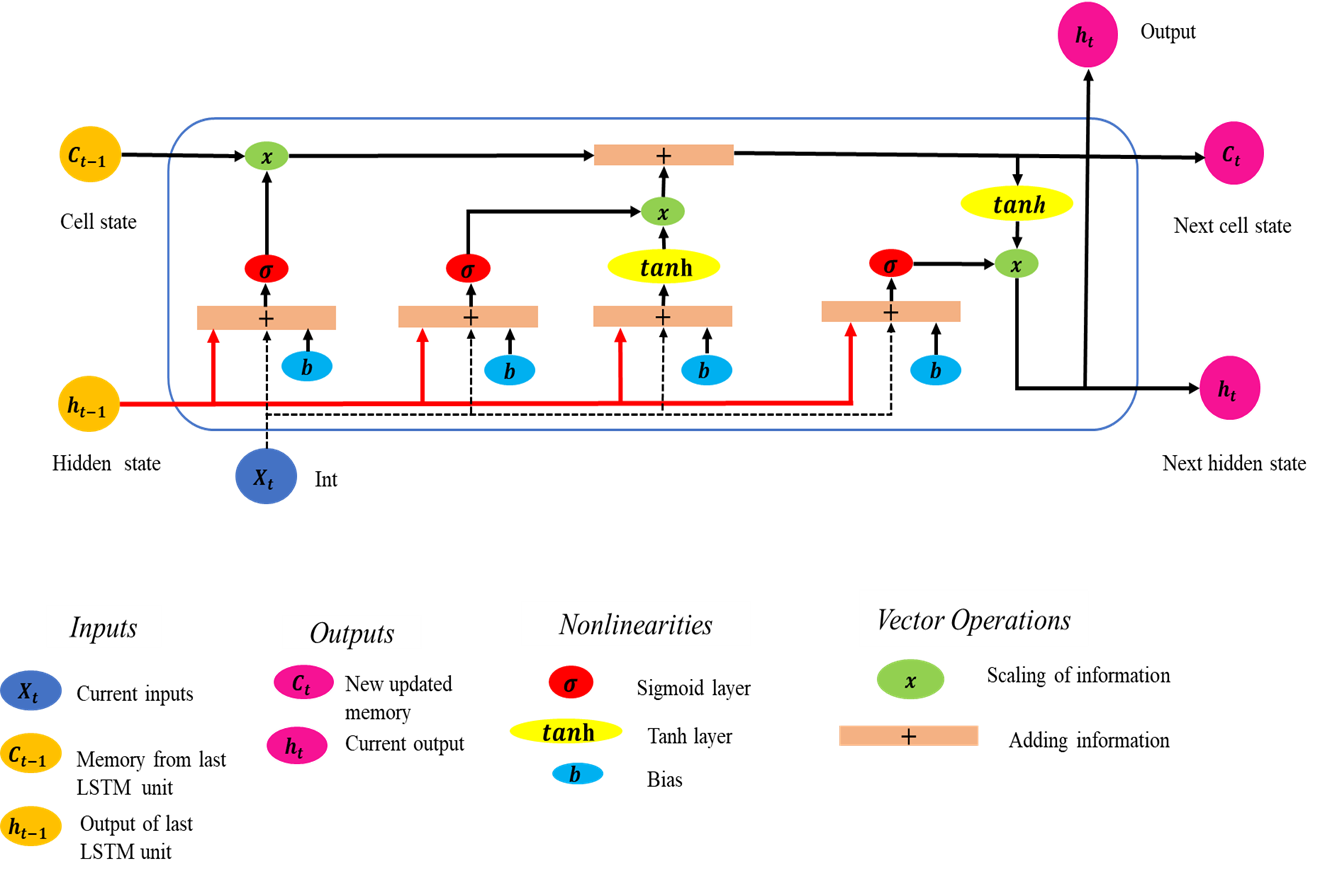 |
| --- |

Appendix C: The main structure of the LSTM model.

| S1 station |
| --- |
| S2 station |

Appendix D: Partial autocorrelation values for studied stations used to identify the best inputs for predictive models.

|  **First half (1965-1991)**  **Second half (1966-1991)** |
| --- |

Appendix E: Monthly drought trends at S1 station using traditional methods: challenges in detecting trends due to fluctuating in the nature of drought data.
